# Oncogenicity Variant Interpreter (OncoVI) Supports Harmonized Somatic Variant Interpretation in Precision Oncology

**DOI:** 10.1016/j.jmoldx.2026.03.004

**Published:** 2026-04-03

**Authors:** Maria Giulia Carta, Lars Tögel, Annett Hölsken, Christoph Schubart, Heinrich Sticht, Robert Stöhr, Silvia Spoerl, Norbert Meidenbauer, Arndt Hartmann, Paolo Magni, Florian Haller, Fulvia Ferrazzi

**Affiliations:** ∗ Institute of Pathology, University Hospital Erlangen, Friedrich-Alexander-Universität Erlangen-Nürnberg, Erlangen, Germany; † Comprehensive Cancer Center Erlangen-EMN, Erlangen, Germany; ‡ Bavarian Cancer Research Centre, Erlangen, Germany; § Center for Personalized Medicine, Erlangen, Germany; ¶ Department of Electrical, Computer and Biomedical Engineering, University of Pavia, Pavia, Italy; ‖ Institute of Biochemistry, Friedrich-Alexander-Universität Erlangen-Nürnberg, Erlangen, Germany; ∗∗ Department of Internal Medicine 5, Hematology and Oncology, University Hospital Erlangen, Friedrich-Alexander-Universität Erlangen-Nürnberg, Erlangen, Germany; †† Department of Nephropathology, University Hospital Erlangen, Friedrich-Alexander-Universität Erlangen-Nürnberg, Erlangen, Germany

## Abstract

Accurate and reproducible interpretation of somatic variants is fundamental for therapy decision-making in patients with cancer. To harmonize and automate oncogenicity classification, Oncogenicity Variant Interpreter (OncoVI), an open-source, Python-based implementation of the Clinical Genome Resource/Cancer Genomics Consortium/Variant Interpretation for Cancer Consortium oncogenicity guidelines, was developed. For each of the guideline criteria, the textual descriptions were interpreted, and publicly available resources were identified to be used as reference. Starting from the genomic coordinates of a variant, OncoVI automatically performs functional annotation, collects relevant evidence from the integrated resources, evaluates each criterion, and provides a final oncogenicity classification. OncoVI achieved an accuracy of 80% on a gold standard set of 93 somatic variants provided by the guidelines, with a sensitivity of 88% for oncogenic/likely oncogenic variants. When applied to a real-world set of 7802 variants from 557 participants previously evaluated by the Molecular Tumor Board (MTB) Erlangen, OncoVI showed 79% concordance with the prior MTB assessment of variant impact on protein function. In addition, expert reassessment of 135 MTB variants, conducted in accordance with the oncogenicity guidelines, further confirmed both the validity of OncoVI implementation and the appropriateness of the identified resources. Taken together, OncoVI provides significant support for the harmonized and reproducible oncogenicity classification of somatic variants across institutions.

## Key Points

• Accurate oncogenicity classification is crucial, but it remains inconsistent across institutions.
• No tools currently automate all the guidelines for standardized oncogenicity classification.
• Oncogenicity Variant Interpreter (OncoVI) automates the evaluation of oncogenicity guidelines using public reference resources.
• OncoVI enables consistent oncogenicity classification and promotes guideline adoption.

Establishing precision oncology as routine practice requires the identification of patient-specific tumor variants, based on comprehensive molecular profiling of cancer cell DNA, and the precise, reproducible classification of the oncogenicity of somatic variants in the context of the disease. Nowadays, tumor molecular profiling is facilitated by accessible and affordable next-generation sequencing technologies.^1^, ^2^, ^3^, ^4^, ^5^, ^6^, ^7^ The interpretation of the pathogenicity of a somatic variant with respect to neoplastic diseases (oncogenicity) defines its role in tumor initiation and progression.^8^ This process requires three fundamental steps: functional annotation, collection of biomedical evidence, and final classification. Functional annotation relies on tools able to predict the potential effect of variants on gene transcripts and protein sequences, such as the Variant Effect Predictor (VEP),^9^ ANNOVAR,^10^ and SnpEff.^11^ This task is complemented by the integration of evidence supporting the variant involvement in tumor development (eg, population data, biomedical literature, and *in silico* predictions). Tools such as VarSome^12^ and cBio Cancer Genomics Portal^13^ integrate and display these different data in an intuitive way, but leave the evaluation of these variant-associated evidence (variant interpretation) to the user. To support this laborious task for genomic variants in cancer, databases containing curated classifications have been developed (eg, the Clinical Interpretation of Variants in Cancer,^14^ the Oncology Knowledge Base (OncoKB),^15^ and the Database of Curated Mutations^16^). However, these databases contain clinical interpretations predominantly limited to well-characterized variants and exhibit differences in the standards describing genes, variants, and diseases.^17^ In addition, tools such as the Molecular Tumor Board Portal and the Cancer Genome Interpreter (CGI) generate interactive reports by leveraging computational methods and publicly available knowledge.^18^^,^^19^ However, the variant classifications provided by these tools do not follow standardized guidelines.

To increase the consistency of variant interpretation between different institutions and assessors, in 2022, three consortia have published the Clinical Genome Resource/Cancer Genomics Consortium/Variant Interpretation for Cancer Consortium oncogenicity guidelines, the so far only internationally recognized guidelines for the classification of oncogenicity of somatic variants in cancer.^8^ On the basis of a standard operating procedure (SOP), including 12 and 5 criteria for oncogenic and benign effect, respectively, each somatic variant is evaluated, and a score is calculated according to a point-based system. This variant-specific score assigns the variant to one of five possible classes (ie, oncogenic, likely oncogenic, variant of uncertain significance (VUS), likely benign, or benign). In the SOP, all criteria are provided as textual indications only; and for some criteria, a careful interpretation by the user is essential. Furthermore, the authors provide recommendations for the use of resources for some, but not all, criteria. The Personal Cancer Genome Reporter tool provides an oncogenicity classification of somatic variants based on these guidelines.^20^ However, the Personal Cancer Genome Reporter does not implement all criteria, and the scoring thresholds for classification have been modified from the SOP, with unknown impact on the final oncogenicity classification.

Here, a further step toward the harmonization of the interpretation of somatic variants is taken by presenting Oncogenicity Variant Interpreter (OncoVI), a tool providing an automated evaluation of the oncogenicity guidelines based on the point-based system proposed by Horak et al.^8^ OncoVI is incorporated in a broader framework that i) performs functional annotation of genomic alterations, ii) collects the available evidence from the interrogated resources, and iii) evaluates each criterion, providing a final classification of oncogenicity.

## Materials and Methods

### Implementation of the Oncogenicity Criteria in OncoVI

**Figure 1:**
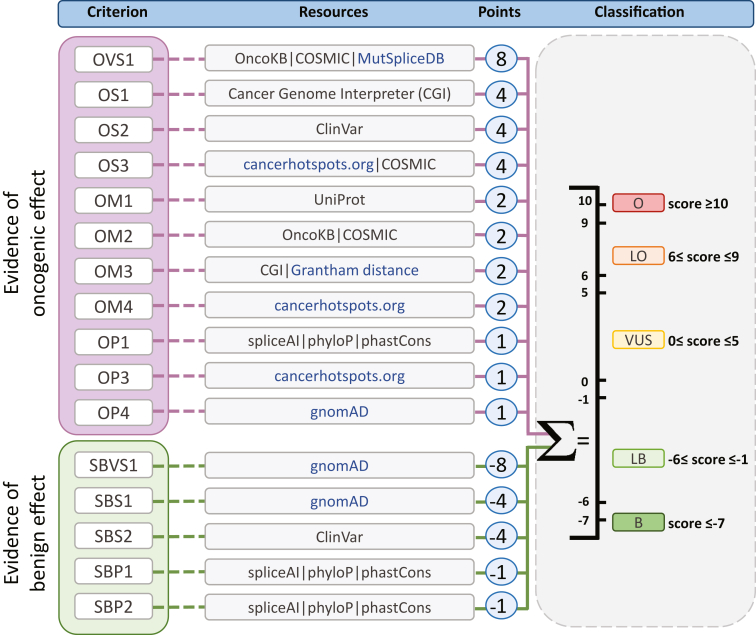
Workflow of OncoVI. Implemented criteria in OncoVI [11 and 5 criteria for evidence of oncogenic (O) and benign (B) effect, respectively], public resources used to assess each criterion, the points associated with it, and the classification of oncogenicity into one of five classes on the basis of the variant-specific score. This score is obtained as the sum of the points associated with the criteria triggered by OncoVI for the variant and the corresponding classification is as follows: score ≥ 10: oncogenic, 6 ≤ score ≤ 9: likely oncogenic (LO), 0 ≤ score ≤ 5: variant of uncertain significance (VUS), –6 ≤ score ≤ –1: likely benign (LB), and score ≤ –7: benign. Resources indicated in blue text were suggested by the standard operating procedure, and resources in black text were identified by the authors of this study. CGI, Cancer Genome Interpreter; COSMIC, Catalogue of Somatic Mutations in Cancer; gnomAD, Genome Aggregation Database; OM1, oncogenic moderate-1 (2 points); OM2, oncogenic moderate-2 (2 points); OM3, oncogenic moderate-3 (2 points); OM4, oncogenic moderate-4 (2 points); OncoKB, Oncology Knowledge Base; OP1, oncogenic supporting-1 (1 point); OP3, oncogenic supporting-3 (1 point); OP4, oncogenic supporting-4 (1 point); OS1, oncogenic strong-1 (4 points); OS2, oncogenic strong-2 (4 points); OS3, oncogenic strong-3 (4 points); OVS1, oncogenic very strong-1 (8 points); phastCons, phastCons100way_vertebrate_rankscore; phyloP, phyloP100way_vertebrate_rankscore; SBP1, somatic benign supporting-1 (–1 point); SBP2, somatic benign supporting-2 (–1 point); SBS1, somatic benign strong-1 (–4 points); SBS2, somatic benign strong-2 (–4 points); SBVS1, somatic benign very strong-1 (–8 points).

All SOP criteria were implemented in OncoVI, except for the Oncogenic Supporting-2 (1 point; “Somatic variant in a gene in a malignancy with a single genetic etiology”) criterion, for which an appropriate resource could not be identified (Figure 1).

The first step of the implementation required interpreting the textual indication provided by the SOP. Then, the resources to use as reference for each criterion were either adopted from those suggested in the SOP or identified from publicly available databases when no suggestion by the SOP was provided. On the basis of an IF-ELSE implementation, OncoVI evaluates the resource used as reference for each criterion and decides whether to trigger the criterion. The next section details the rationale used to interpret the textual indication associated with each criterion, as well as the implementation in OncoVI, and the associated resources identified as reference.

The code for the implementation of the oncogenicity guidelines in OncoVI is available at *https://github.com/MGCarta/oncovi* (last accessed April 24, 2025).

### Criteria for Evidence of Oncogenicity of Somatic Variants

#### OVS1 Criterion

Oncogenic very strong-1 (OVS1; 8 points) description is “Null variants (nonsense, frameshift, canonical ±1 or 2 splice sites, initiation codon, single-exon or multiexon deletion) in a bona fide tumour suppressor genes.” Null variants were defined relying on the calculated consequences by VEP.^9^ “Nonsense” corresponded to “stop_gained”, “frameshift” corresponded to “frameshift_variant”. “Stop_lost” variants were included because they may have impact on protein functionality (eg, by disrupting protein stability or by altering overall tertiary protein structure).^21^ “Canonical ±1 or 2 splice sites” corresponded to either “splice_donor_variant” or “splice_acceptor_variant”. In addition, “splice_region_variants” occurring within 1 to 4 bp of exon-intron boundaries were included, thereby increasing the splice region window, because alterations in these proximal regions may result in aberrant transcript isoforms.^22^ “Initiation codon” corresponded to “start_lost”. “Single-exon or multiexon deletion” was not implemented, given that VEP does not provide any consequence representing these variants. The set of bona fide tumor suppressor genes (TSGs) was generated as the intersection of two resources: the Cancer Gene Census of the Catalogue of Somatic Mutations in Cancer (COSMIC)^23^ and OncoKB.^15^ From the Cancer Gene Census, genes with “Tier” equal to “1” and “Role in Cancer” equal to “TSG” were used (*https://cancer.sanger.ac.uk/cosmic/download/cosmic*, Census_allWed_May_1_17_27_56_2024.csv, v99, GRCh38, last accessed May 1, 2024). From OncoKB, genes in the Cancer Gene List with “Is Tumor Suppressor Gene” equal to “Yes” were used (*https://www.oncokb.org/cancer-genes*, cancerGeneList.tsv, last accessed April 29, 2024). For variants not triggering OVS1, MutSpliceDB^24^ is consulted (*https://brb.nci.nih.gov/cgi-bin/splicing/splicing_main.cgi*, MutSpliceDB_BRP_2024-02-06.csv, last accessed February 6, 2024).

#### OS1 Criterion

Oncogenic strong-1 (OS1; 4 points) description is “Same amino acid change as a previously established oncogenic variant (using this standard) regardless of nucleotide change.” This criterion requires variants previously classified according to the SOP. However, because this set of variants is not available when applying the guidelines for the first time, as substitute for “previously established oncogenic variant” OncoVI relies on a publicly available database of validated oncogenic variants (namely, the Catalog of Validated Oncogenic Mutations of the CGI database, *https://www.cancergenomeinterpreter.org/mutations*, last accessed February 1, 2024).^18^ All variants regardless of the context were considered.

#### OS2 Criterion

Oncogenic strong-2 (OS2; 4 points) description is “Well-established *in vitro* or *in vivo* functional studies, supportive of an oncogenic effect of the variant.” ClinVar application programming interface (API) was used as reference for OS2.^25^ Variants with a clinical significance “Pathogenic” or “Likely pathogenic” or within a curated list of clinical significances (ie, "Affects; risk factor", "association", "association; drug response; risk factor", "association; risk factor", "confers sensitivity", "confers sensitivity; other", "drug response", "drug response; other", "drug response; risk factor", "other; risk factor", "protective; risk factor", "risk factor"), and a submission method with the presence of functional studies (ie, “Practice guideline”, “Reviewed by expert panel”, “Criteria provided, multiple submitters, no conflicts”, “Criteria provided, single submitter”) were considered. This choice was mainly dictated by the fact that ClinVar variants from *in vivo* and *in vitro* studies represent only the 0.2% (6891 of 3,462,730; *https://ftp.ncbi.nlm.nih.gov/pub/clinvar/tab_delimited*, submission_summary.txt.gz, last accessed April 12, 2025).

#### OS3 Criterion

Oncogenic strong-3 (OS3; 4 points) description is “Located in one of the hotspots in cancerhotspots.org with at least 50 samples with a somatic variant at the same amino acid position, and the same amino acid change count in cancerhotspots.org in at least 10 samples”; and for this criterion, the resource suggested by the SOP was used (ie, cancerhotspots.org that contains well-known hotspot variants, *https://www.cancerhotspots.org/#/home*, last accessed September 26, 2024).^26^^,^^27^ In addition, to ensure a comprehensive assessment of hotspot variants, COSMIC data were included (*https://cancer.sanger.ac.uk/cosmic/download/cosmic*, COSMIC Census Genes Mutations, last accessed April 9, 2024, v99; COSMIC Cancer Mutation Census, last accessed May 7, 2024, v99), considering variants found in at least 50 tumor samples, with at least 10 showing the exact nucleotide change.

#### OM1 Criterion

Oncogenic moderate-1 (OM1; 2 points) description is “Located in a critical and well-established part of a functional domain.” For OM1, an extended investigation into the biological characteristic addressed by the criterion was needed. For example, in OncoVI implementation, OM1 addresses variants located in a functional domain of the protein. Indeed, a functional domain, by definition, folds and functions independently from the rest of the polypeptide chain, and it was therefore hypothesized that variants affecting protein domains are more likely to disrupt the protein function. To this aim, human protein domains from UniProt were used (*https://ftp.ebi.ac.uk/pub/databases/uniprot/current_release/knowledgebase/genome_annotation_tracks/UP000005640_9606_beds*, UP000005640_9606_domain.bed, release 2024_02, last accessed April 16, 2024).^28^ UniProtKB_AC (accession identifiers) were extracted and converted into Human Genome Organisation^29^ gene symbols, via the webservice provided by UniProt, to be used as identifiers in the implementation. OncoVI verifies whether UniProt includes a protein encoded by the gene affected by the variant under evaluation. If the nucleotide triplet affected by the variant belongs to a functional domain, then OM1 is triggered, otherwise not.

#### OM2 Criterion

Oncogenic moderate-2 (OM2; 2 points) description is “Protein length changes as a result of in-frame deletions/insertions in a known oncogene (OG) or tumor suppressor gene or stop-loss variants in a known tumor suppressor gene.” For “in-frame deletions/insertions” VEP annotated consequences “inframe_deletion” and “inframe_insertion” were considered. To generate TSG and OG groups, the union of OncoKB Cancer Gene List and COSMIC Cancer Gene Census genes, regardless of the tier, was used.

#### OM3 Criterion

Oncogenic moderate-3 (OM3; 2 points) is “Missense variant at an amino acid residue where different missense variant determined to be oncogenic (using this standard) has been documented. Amino acid difference should be greater or at least approximately the same as for missense change determined to be oncogenic.” For missense variants, the VEP annotated consequence “missense_variant” was considered. The Catalog of Validated Oncogenic Mutations of the CGI database was used as substitute for “previously established oncogenic variant.” To measure the difference between reference and alternate amino acids, the Grantham distance was used.^30^

#### OM4 Criterion

Oncogenic moderate-4 (OM4; 2 points) is “Located in one of the hotspots in cancerhotspots.org with <50 samples with a somatic variant at the same amino acid position, and the same amino acid change count in cancerhotspots.org is at least 10.” As for OS3 criterion, cancerhotspots.org was used (*https://www.cancerhotspots.org/#/home*, last accessed September 26, 2024).

#### OP1 Criterion

Oncogenic supporting-1 (OP1; 1 point) description is “All used lines of computational evidence support an oncogenic effect of a variant.” The computational algorithms consulted by OncoVI to trigger OP1, somatic benign supporting-1 (SBP1), and somatic benign supporting-2 (SBP2) are those cited by the SOP to predict conserved elements across species and splicing effect [ie, predictive scores from phyloP100way_vertebrate_rankscore (phyloP)^31^ and phastCons100way_vertebrate_rankscore (phastCons)].^32^ These scores were retrieved on the basis of the VEP plugin dbNSFP (database of non synonymous functional prediction, version 4.5a), and scores from spliceAI^33^ were retrieved via the VEP plugin spliceAI. Variants with a phyloP or phastCons score ≥0.5 or a spliceAI score equal to “PASS” were considered to trigger the criterion.

#### OP3 Criterion

Oncogenic supporting-3 (OP3; 1 point) description is “Located in one of the hotspots in cancerhotspots.org and the particular amino acid change count in cancerhotspots.org is below 10.” As for OS3 and OM4, cancerhotspots.org was used (*https://www.cancerhotspots.org/#/home*, last accessed September 26, 2024).

#### OP4 Criterion

Oncogenic supporting-4 (OP4; 1 point) description is “Absent from controls (or at an extremely low frequency) in gnomAD.” Genome Aggregation Database (gnomAD) exome (v2.1.1) and genome frequency data (v3.1.2)^34^ were used as populated via VEP. The implementation of this criterion required choosing a threshold for the gnomAD population frequency [ie, variants absent in gnomAD exome or genome frequency data or with a gnomAD exome or gnomAD genome frequency value 0.01 trigger OP4].

### Criteria for Evidence of Benign Effect of Somatic Variants

#### SBVS1 Criterion

Somatic benign very strong-1 (SBVS1; –8 points) description is “Minor allele frequency is >5% in gnomAD in any 5 general continental populations: African, East Asian, European (non-Finnish), Latino, and South Asian.” The criterion is triggered by variants with a gnomAD frequency ≥0.05 in at least one of the five populations.

#### SBS1 Criterion

Somatic benign strong-1 (SBS1; –4 points) description is “Minor allele frequency is >1% in gnomAD in any 5 general continental populations: African, East Asian, European (non-Finnish), Latino, and South Asian.” The criterion is triggered by variants with a gnomAD frequency ≥0.01 in at least one of the five populations.

#### SBS2 Criterion

Somatic benign strong-2 (SBS2; –4 points) description is “Well-established *in vitro* or *in vivo* functional studies show no oncogenic effects.” Variants with a clinical significance “Benign” or “Likely benign” and a review status assuming a submission method reporting the presence of functional studies trigger SBS2.

#### SBP1 Criterion

SBP1 (–1 point) description is “All used lines of computational evidence suggest no effect of a variant.” phyloP and phastCons <0.5 or spliceAI score equal to “FAIL” trigger SBP1.

#### SBP2 Criterion

SBP2 (–1 point) description is “A synonymous (silent) variant for which splicing prediction algorithms predict no effect on the splice consensus sequence nor the creation of a new splice site and the nucleotide is not highly conserved. For synonymous variants, VEP calculated consequence equal to “synonymous_variant” were considered. phyloP and phastCons scores <0.5 or spliceAI score equal to “FAIL” trigger SBP2.

### Standard Operating Procedure Data Set

The SOP data set contains 93 variants, retrieved from Supplementary Tables 1 and 3 (see Horak et al^8^). For the 70 missense variants in Supplementary Table 3 (see Horak et al^8^), detailed genomic information (DNA change, chromosome, position, strand, reference, and alternate bases) were provided for both genome builds 37 and 38. The remaining 23 variants from Supplementary Table 1 (see Horak et al^8^) lacked these details, which were manually derived from the DNA change. For all variants, the triggered criteria, variant-specific scores, and oncogenicity classification were collected (Supplemental Table S1).

### Molecular Tumor Board Data Set

The real-world Molecular Tumor Board (MTB) data set comprises 7802 unique somatic variants across 489 genes, identified in a cohort of 557 individuals with various tumor diagnoses, enrolled in the MTB of the Comprehensive Cancer Center Erlangen-EMN (Erlangen, Germany) from February 2022 to February 2024. Formalin-fixed, paraffin-embedded patient tissue was sequenced on a NextSeq500 using the TruSight Oncology 500 assay (Illumina, Inc., San Diego, CA), which investigates 523 cancer-related genes. Sequencing raw data (bcl files) were processed with the TruSight Oncology 500 version 2.2 Local App, deployed via Docker on a remote Ubuntu 20.04.6 long-term support server. The pipeline performs demultiplexing, FASTQ generation, DNA/RNA alignment to the human reference genome (University of California, Santa Cruz, hg19), and calling of single and multiple nucleotide variants, copy number variants, fusions, and splice variants. Genomic variant positions per individual were provided in a variant call format (VCF) file called MergedSmallVariants.genome.vcf. Three expert cancer biologists (L.T., A.Hö., and C.S.) assessed variants in terms of their effect on the wild-type protein function and categorized them into five classes (ie, pathogenic, likely pathogenic, variant of uncertain significance, likely benign, or benign). Later, the multidisciplinary MTB team evaluated variants for their actionability.

The study was conducted in accordance with the Declaration of Helsinki and approved by the Ethics Committee of the Friedrich-Alexander University Erlangen-Nuremberg (100_17 B from April 7, 2017, addendum from July 27, 2021).

### Validation Data Set

The validation data set of 135 somatic variants was generated from the MTB data set to validate the implementation of the oncogenicity criteria in OncoVI. These variants were chosen *a posteriori* based on the analysis of the entire MTB data set (Supplemental Table S2). More specifically, the validation data set was generated on the basis of the obtained confusion matrix between MTB and OncoVI classifications by considering: all the variants with the strongest disagreement (ie, pathogenic/likely pathogenic (P/LP) MTB variants classified as benign/likely benign (B/LB) by OncoVI and B/LB MTB variants classified as oncogenic/likely oncogenic (O/LO) by OncoVI); and randomly chosen variants from the confusion matrix cells corresponding to OncoVI classification as VUS and O/LO, with a higher number from the MTB P/LP variants classified as VUS by OncoVI. The same three expert cancer biologists, who curated the classification of the MTB data set, reclassified the validation data set according to the oncogenicity criteria. To this aim, the 135 variants were randomly divided into three sets, and each expert was provided with the list of the resources used by OncoVI for each criterion.

### ClinVar Data Set

The ClinVar data set includes 691 somatic variants with available oncogenicity classification in ClinVar (*https://ftp.ncbi.nlm.nih.gov/pub/clinvar/tab_delimited*, variant_summary.txt.gz, last accessed April 12, 2025*).* For these variants, oncogenicity was calculated by the National Center for Biotechnology Information based on submitter data following the oncogenicity guidelines.^8^ Chromosome, PositionVCF, ReferenceAlleleVCF, and AlternateAlleleVCF in the hg38 genome build were used to generate the input VCF file for annotation with the VEP-based in-house pipeline. ClinVar oncogenicity classification was considered as ground truth to evaluate OncoVI performance on the ClinVar data set.

### Variant Annotation via the VEP-Based In-House Pipeline

Functional annotation of the variants in the SOP (Supplemental Table S3), MTB (Supplemental Table S4), and ClinVar (Supplemental Table S5) data sets was performed, relying on a custom Python-based pipeline (Python version 3.8.8; *https://www.python.org*). The pipeline runs in a dedicated conda environment on a remote server based on Ubuntu 20.04.6 long-term support operating system. The pipeline takes the genomic positions of the variants as input. For the SOP and ClinVar data sets, the coordinates in the hg38 reference genome build were used. For the variants of the MTB data set, genomic coordinates were obtained by uplifting the original hg19-based VCFs (ie, MergedSmallVariants.genome.vcf) to the genome reference build hg38 via the LiftOverVcf function of the picard package version 3.0.0 (*https://anaconda.org/channels/bioconda/packages/picard/overview*).

First, the Python-based pipeline filters only genomic coordinates with an alternate allele different from the reference allele, then only variants with FILTER equal to PASS or Blacklist are retained using the function filter_vcf of the VcfFilterPy package (*https://github.com/superDross/VcfFilterPy*, last accessed February 15, 2024). Afterwards, the variants are functionally annotated with the VEP from Ensembl (VEP version 111.0, January 2024) using GRCh38 and Reference Sequence Database^35^ as preferred cache. Two VEP plugins, dbNSFP version 4.5a and spliceAI, were used to retrieve scores from predictive algorithms. After annotation, the filter_vep tool removed from the VCF the variants without consequence and/or with a global gnomAD population frequency ≥0.025. In addition, only variants with a VEP-annotated coding consequence were retained. Furthermore, for variants for which multiple transcripts were annotated, the canonical transcript according to the Matched Annotation from NCBI and EBI (MANE) project^36^ was favored.

Eventually, the pipeline interrogates several knowledgebases to retrieve existing biomedical information of the somatic variants. In the case of ClinVar, only the clinical significance and review status from ClinVar were retrieved via the ClinVar API. As final output, the pipeline provides the list of annotated variants that underwent the above-mentioned steps [as comma-separated values (csv) and Microsoft (Redmond, WA) Excel format].

### Data Analysis and Statistical Analysis

All statistical analyses were performed in R version 4.1.2/Bioconductor version 3.13 (*https://www.r-project.org*).^37^^,^^38^ Spearman correlations were computed using cor.test() from the stats package. Contingency tables were generated with caret version 6.0.94. Odds ratios were calculated to test the association between OncoVI triggered criteria and the correct classification of oncogenic/likely oncogenic variants of the SOP data set. Statistical significance was tested via a two-sided Fisher exact test, with *P* ≤ 0.05 considered statistically significant. All plots were generated using ggplot2 version 3.5.1 and ggalt version 0.4.0 (*https://cran.r-project.org*). OncoPrints were produced with ComplexHeatmap version 2.13.1 (*https://cran.r-project.org*), and alluvial plots were produced with ggalluvial version 0.12.5 (*https://cran.r-project.org*).

## Results

OncoVI is a Python-based tool automating the evaluation of the oncogenicity criteria (Figure 1). Each criterion was interpreted whenever its biological meaning resulted unclear from the SOP textual indication. Then, the resources suggested by the SOP were adopted or, when there was no suggestion, identified from publicly available databases. Finally, using an IF-ELSE logic for each criterion, OncoVI assigned the associated points and provided the oncogenicity classification based on the calculated variant-specific score. For example, the missense variant *IDH1*:p.Arg132His (GRCh38, 2: 208248388C>T) according to the CGI database has been described in acute myeloid leukemia, brain, malignant astrocytoma (OS1, +4), was reported in ClinVar as pathogenic/likely pathogenic (OS2, +4), was present in gnomAD at frequency <0.01 (OP4, +1), and was predicted by *in silico* algorithms with score ≥0.5 (OP1, +1). Collectively, the variant was classified as oncogenic by OncoVI with a variant-specific score of 10.

### Performance of OncoVI on the Standard Operating Procedure Data Set

To assess the validity of its implementation and used resources, OncoVI was tested on the SOP data set, a data set of 93 somatic variants provided by the guideline authors. Of these, according to SOP authors, 50 variants occurred in six OGs, 31 in three bona fide TSGs, and 12 in genes whose role in cancer is context dependent. SOP oncogenicity classification had assigned the variants to five classes as follows: oncogenic: 14, likely oncogenic: 29, VUS: 38, likely benign: 6, and benign: 6 (Supplemental Table S1). Functional annotation using the in-house VEP-based pipeline revealed that most variants were missense (*n* = 71), followed by truncating alterations, including nonsense and frameshift variants (*n* = 9). Four variants were in splice sites, whereas five variants were located in upstream gene regions; the remaining variants comprised intronic, untranslated region, synonymous, or in-frame changes (Supplemental Table S3). The three most frequently annotated genes were *EZH2* (*n* = 12), *RB1* (*n* = 11), and *BRAF* (*n* = 10).

**Figure 2:**
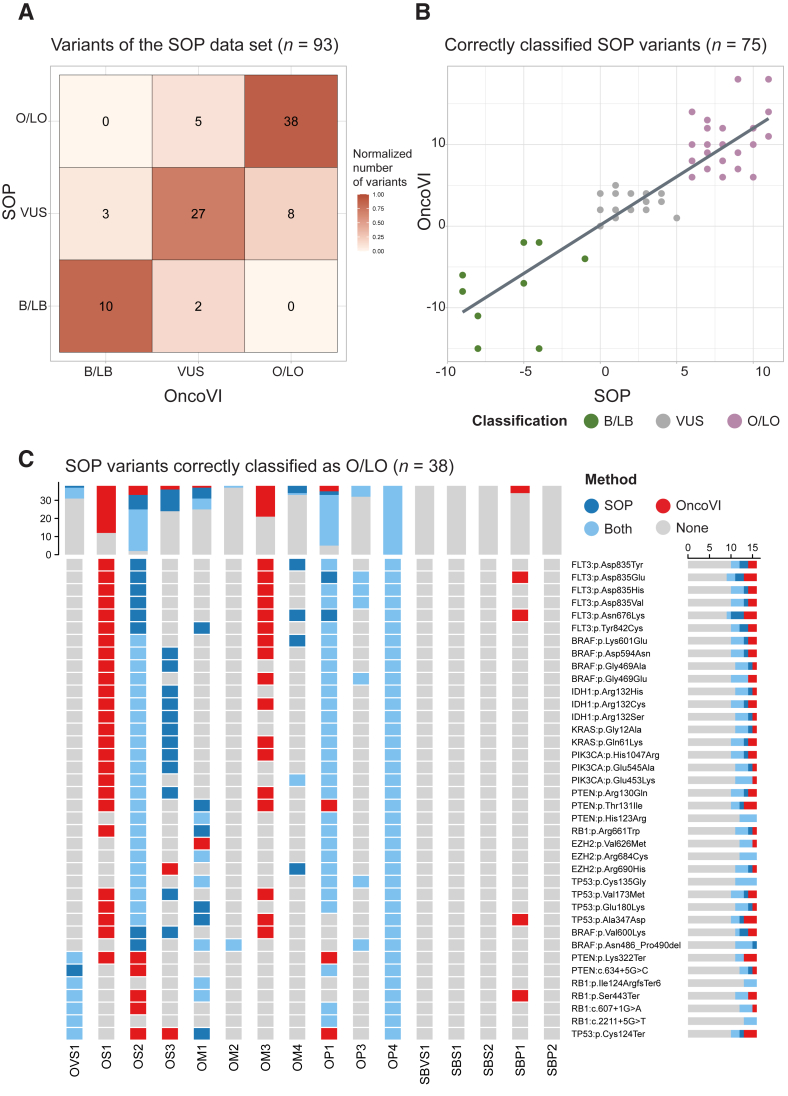
Results of OncoVI on the standard operating procedure (SOP) data set. **A:** Confusion matrix of the agreement between the SOP and OncoVI in classifying the variants of the SOP data set. The color scale indicates the normalized number of variants [ie, the ratio (calculated by row) between the number of variants of each cell and the total number of variants]. **B:** Scatterplot between the points assigned by the SOP and the points assigned by OncoVI for the 75 correctly classified variants. **C:** Oncoprint of the 38 variants of the SOP data set correctly classified as O/LO. Each row corresponds to one variant, and each column corresponds to an assessed criterion. The color of the cell indicates whether a criterion was triggered by the SOP only (dark blue), OncoVI only (red), both (light blue), or none of the two (gray). The top bar plot shows the sum of cells of each color calculated across all variants, and the bar plot on the right shows the sum across all criteria. B/LB, benign/likely benign; OM1, oncogenic moderate-1 (2 points); OM2, oncogenic moderate-2 (2 points); O/LO, oncogenic/likely oncogenic; OM3, oncogenic moderate-3 (2 points); OM4, oncogenic moderate-4 (2 points); OP1, oncogenic supporting-1 (1 point); OP3, oncogenic supporting-3 (1 point); OP4, oncogenic supporting-4 (1 point); OS1, oncogenic strong-1 (4 points); OS2, oncogenic strong-2 (4 points); OS3, oncogenic strong-3 (4 points); OVS1, oncogenic very strong-1 (8 points); SBP1, somatic benign supporting-1 (–1 point); SBP2, somatic benign supporting-2 (–1 point); SBS1, somatic benign strong-1 (–4 points); SBS2, somatic benign strong-2 (–4 points); SBVS1, somatic benign very strong-1 (–8 points); VUS, variant of uncertain significance.

To evaluate OncoVI performance, variants were grouped into O/LO, B/LB, and VUS classes. Using the SOP oncogenicity classification as ground truth, OncoVI achieved 81% accuracy (*n* = 75/93 correctly classified variants), with a sensitivity of 88% (*n* = 38/43) and 83% (*n* = 10/12) for the O/LO and B/LB class, respectively (Figure 2A). When considering the 75 correctly classified variants, a strong positive correlation (*r* = 0.87, *P* < 2.2 × 10^–^^16^) between the variant-specific scores assigned by the SOP and by OncoVI was observed (Figure 2B). Furthermore, OncoVI usually assigned higher scores than the SOP in variants correctly classified as O/LO (Supplemental Figure S1A) and in variants classified as VUS (Supplemental Figure S2C), and lower scores in correctly classified B/LB variants (Supplemental Figure S2A). When evaluating OncoVI performance stratified by gene classes (OGs versus TSGs), higher accuracy for variants in TSGs compared with OGs was observed (87.1% versus 82.0%) (Supplemental Table S6). When stratified by variant functional consequence, accuracy was higher for missense than truncating variants (83.1% versus 77.7%) (Supplemental Table S6).

To further assess the choice of the chosen resources, first the most frequently triggered criteria by OncoVI in the 75 correctly classified variants were investigated. The results showed that very strong and strong criteria contributed the most to OncoVI correct classification of oncogenic and benign variants (Supplemental Figure S1B and Supplemental Figure S2B), whereas for variants classified as VUS, OncoVI triggered only supporting and moderate oncogenic criteria (Supplemental Figure S2D). In addition, OS1 (odds ratio, 11.8; *P* = 0.013) and OS2 (odds ratio, 6.29; *P* = 0.036) were the oncogenic criteria most strongly associated with a correct classification, whereas no benign criterion showed association (Table 1).

**Table 1:** OR of the Association between the Triggering of Criteria and the Correct Classification of Oncogenic or Likely Oncogenic Variants of the SOP Data Set

Criterion	OR	95% CI	*P* value
OVS1	1.27	0.12–66.78	1
OS1	11.8	1.24–591.71	***0.013***
OS2	6.29	0.87–75.17	***0.036***
OS3	0.5	0.03–29.75	0.488
OM1	0.2	0.024–1.41	0.06
OM2	0.33	0.015–21.71	0.39
OM3	4.77	0.51–237.82	0.22
OM4	0.33	0.01–21.71	0.39
OP1	2.912	0.36–21.45	0.33
OP3	1.27	0.12–66.78	1
OP4	6.09	0.07–520.21	0.28
SBVS1	0.16	0.002–14.07	0.28
SBS1	0.16	0.002–14.07	0.28
SBS2	0.16	0.002–14.07	0.28
SBP1	0.86	0.07–47.21	1
SBP2	0.16	0.002–14.07	0.28

Bolded italicized values indicate *P* ≤ 0.05. *P* values are from the Fisher exact test.

OM1, oncogenic moderate-1 (2 points); OM2, oncogenic moderate-2 (2 points); OM3, oncogenic moderate-3 (2 points); OP1, oncogenic supporting-1 (1 point); OP3, oncogenic supporting-3 (1 point); OP4, oncogenic supporting-4 (1 point); OR, odds ratio; OS1, oncogenic strong-1 (4 points); OS3, oncogenic strong-3 (4 points); OVS1, oncogenic very strong-1 (8 points); SBP1, somatic benign supporting-1 (–1 point); SBP2, somatic benign strong-2 (–4 points); SBS1, somatic benign strong-1 (–4 points); SBS2, somatic benign strong-2 (–4 points); SBVS1, somatic benign very strong-1 (–8 points); SOP, standard operating procedure.

To investigate the higher variant-specific scores assigned by OncoVI, the criteria triggered by the SOP and OncoVI for each of the 38 variants correctly classified as O/LO were compared (Figure 2C). The strongest agreement was observed for OP3 (*n* = 6/6), OP4 (*n* = 38/38), OP1 (*n* = 28/33), OVS1 (*n* = 6/7), OM1 (*n* = 6/12), and OS2 (*n* = 23/36). Notably, the SOP activated OS2 based on manual literature search, whereas OncoVI relied on ClinVar pathogenicity. OS1 [strong, 4 points, “Same amino acid change as a previously established oncogenic variant (using this standard) regardless of nucleotide change”] and OM3 [moderate, 2 points, “Missense variant at an amino acid residue where a different missense variant determined to be oncogenic (using this standard) has been documented”] were exclusively triggered by OncoVI because it used an external data set of validated oncogenic variants to define the “previously established oncogenic variants.” Conversely, OS3 was triggered almost exclusively by the SOP (*n* = 12/14) because, according to the guidelines, it is not applicable when OS1 is activated. Overall, these findings confirmed that OncoVI automated implementation of the oncogenicity guidelines closely aligned with SOP manual classification. In addition, OncoVI correct classification appeared to primarily depend on the triggering of strong criteria, supporting the appropriateness of the chosen resources.

### Performance of OncoVI on the Molecular Tumor Board Data Set

To evaluate OncoVI performance in a real-world context, it was applied to 7802 unique somatic variants (MTB data set) previously classified by three expert cancer biologists within the Erlangen MTB. The somatic variants in the MTB cohort were distributed across multiple tumor entities, with the largest number of patients diagnosed with cancers of unknown primary (*n* = 74), followed by pancreatic (*n* = 62), biliary tract (*n* = 49), and colorectal (*n* = 45) carcinomas (Supplemental Figure S3A). Unlike the SOP data set, the MTB data set was larger and more heterogeneous. According to the COSMIC Cancer Gene Census, 3109 variants occurred in TSGs, 1477 occurred in OGs, and 880 occurred in genes with a dual role. The remaining 2336 variants were in genes with an unknown role. Functional annotation using the in-house VEP-based pipeline revealed that most variants were missense (*n* = 6366), followed by truncating alterations, including nonsense, frameshift, and start-loss variants (*n* = 1015). Splice site–related variants accounted for 256 alterations, whereas in-frame insertions/deletions were 164 (Supplemental Table S4). The five most frequently annotated genes were *TP53* (*n* = 193), *APC* (*n* = 152), *LRP1B* (*n* = 151), *FAT1* (*n* = 106), and *ZFHX3* (*n* = 100).

**Figure 3:**
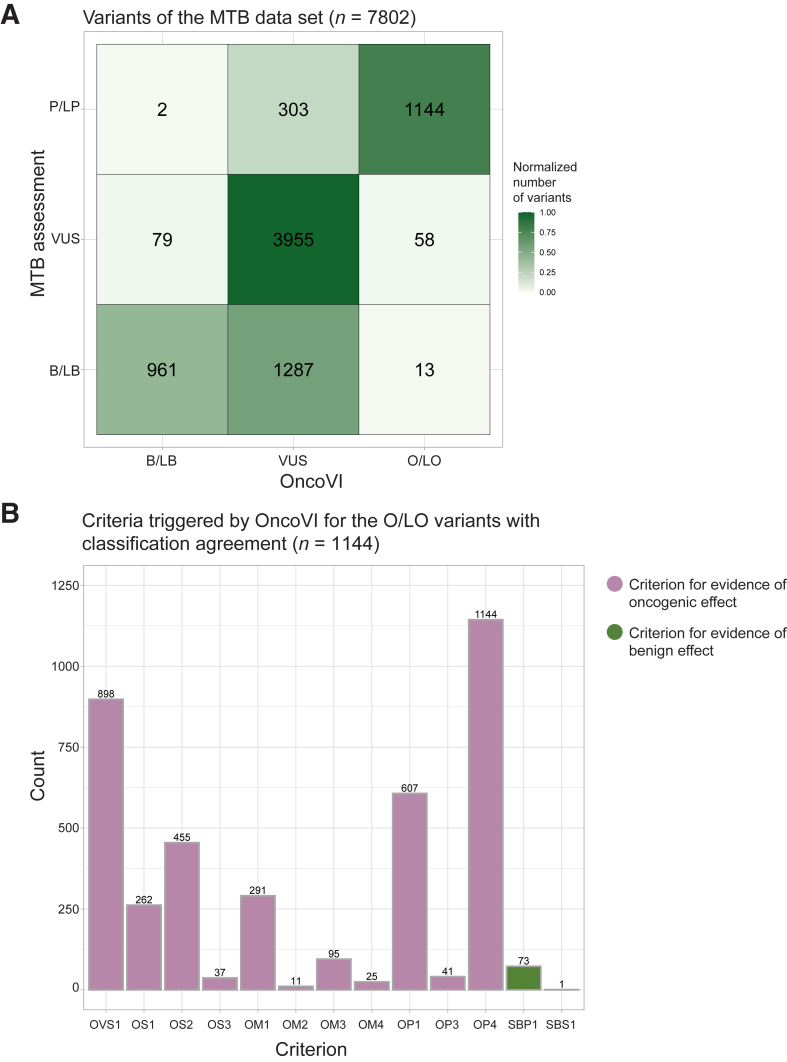
Results of OncoVI on the Molecular Tumor Board (MTB) data set. **A:** Confusion matrix of the agreement between the MTB assessment and OncoVI oncogenicity classification on the 7802 variants of the MTB data set. The color scale indicates the normalized number of variants [ie, the ratio (calculated by row) between the number of variants of each cell and the total number of variants]. **B:** Bar plot of the criteria triggered by OncoVI in the 1144 oncogenic/likely oncogenic (O/LO) variants with classification agreement between MTB and OncoVI assessments. Criteria are sorted according to decreasing corresponding points: OVS1, oncogenic very strong-1 (8 points); OS1, oncogenic strong-1 (4 points); OS2, oncogenic strong-2 (4 points); OS3, oncogenic strong-3 (4 points); OM1, oncogenic moderate-1 (2 points); OM2, oncogenic moderate-2 (2 points); OM3, oncogenic moderate-3 (2 points); OM4, oncogenic moderate-4 (2 points); OP1, oncogenic supporting-1 (1 point); OP3, oncogenic supporting-3 (1 point); OP4, oncogenic supporting-4 (1 point); SBP1, somatic benign supporting-1 (–1 point); SBS1, somatic benign strong-1 (–4 points). B/LB, benign/likely benign; P/LP, pathogenic/likely pathogenic; VUS, variant of uncertain significance.

MTB variants had not been assessed for oncogenicity but for their effect on protein function, and classified as pathogenic, likely pathogenic, VUS, likely benign, or benign (Supplemental Figure S3B and Supplemental Table S7). For the comparison with OncoVI, variants were grouped into P/LP, B/LB, and VUS classes. The agreement between OncoVI oncogenicity classification into O/LO, VUS, and B/LB and the MTB classification into P/LP, VUS, and B/LB was 78% (*n* = 6060/7802 variants), in line with the accuracy observed for the SOP data set (81%; *n* = 75/93). In particular, VUS and B/LB classes had an agreement of 97% (*n* = 3955/4092) and 43% (*n* = 961/2261), respectively, whereas the concordance between OncoVI O/LO and MTB P/LP was 79% (*n* = 1144/1449) (Figure 3A). When evaluating the performance stratified by gene classes (OGs versus TSGs), differently from the SOP data set, higher concordance for variants in OGs compared with TSGs was observed (82.5% versus 76.0%) (Supplemental Table S6). Instead, as in the SOP data set, the performance stratified by functional consequence showed higher concordance for missense variants compared with truncating alterations (78.2% versus 49.4%) (Supplemental Table S6).

The most critical issue in using OncoVI for oncogenicity classification is false-negative oncogenic variants. Two P/LP variants were classified by OncoVI as B/LB, and both triggered SBS2 (strong, –4 points, “Well-established *in vivo* or *in vitro* functional studies showing no oncogenic effect”) because classified in ClinVar as benign/likely benign at the time of submissions (December 2023). In addition, the majority (64%; *n* = 196/303) of the 303 P/LP variants classified as VUS by OncoVI were truncating (*n* = 138) or splice site (*n* = 37) variants, according to the VEP-based pipeline. This is in line with the lower overall agreement observed for truncating variants. Notably, these variants did not occur in bona fide TSGs and, therefore, the OVS1 criterion (8 points for null variants in bona fide TSGs) was not applicable, which most likely is the reason why they were classified as VUS by OncoVI. Thirteen B/LB variants were classified by OncoVI as O/LO because of the frequent activation of OVS1 (very strong, 8 points). Conversely, the MTB classified these 13 variants as B/LB most likely either because of i) their location in protein terminal regions or ii) in a splice region, or iii) for conflicting interpretation of pathogenicity in ClinVar.

The analysis of the most frequently triggered criteria in the 1144 variants with concordance between MTB assessment and OncoVI oncogenicity classification revealed that the top most frequently triggered criteria were as follows: OP4, OVS1, and OP1 (Figure 3B). OVS1 (very strong, 8 points, “Null variants in a bona fide TSG”) was triggered in 79% (*n* = 898/1144) of the variants, reflecting a higher proportion of TSGs than in the SOP data set (16%; *n* = 6/38). Among the 961 B/LB variants, SBS2 (strong, –4 points) was predominant (*n* = 821/961), confirming ClinVar as a suitable reference (Supplemental Figure S4A). SBP1 (*n* = 549), SBS1 (*n* = 496), and SBVS1 (*n* = 149) were also often triggered. In the 3955 variants classified as VUS by both methods, OP4 (*n* = 3955), OP1 (*n* = 2988), and OM1 (*n* = 1759) were the most frequent (Supplemental Figure S4B). Overall, OncoVI oncogenicity classification showed strong concordance with the MTB assessment of the variant effect on protein function and reliably triggered strong oncogenic and benign criteria in correctly classified O/LO and B/LB real-world variants.

### Comparative Analysis with Expert Re-Assessment of the Validation Data Set

**Figure 4:**
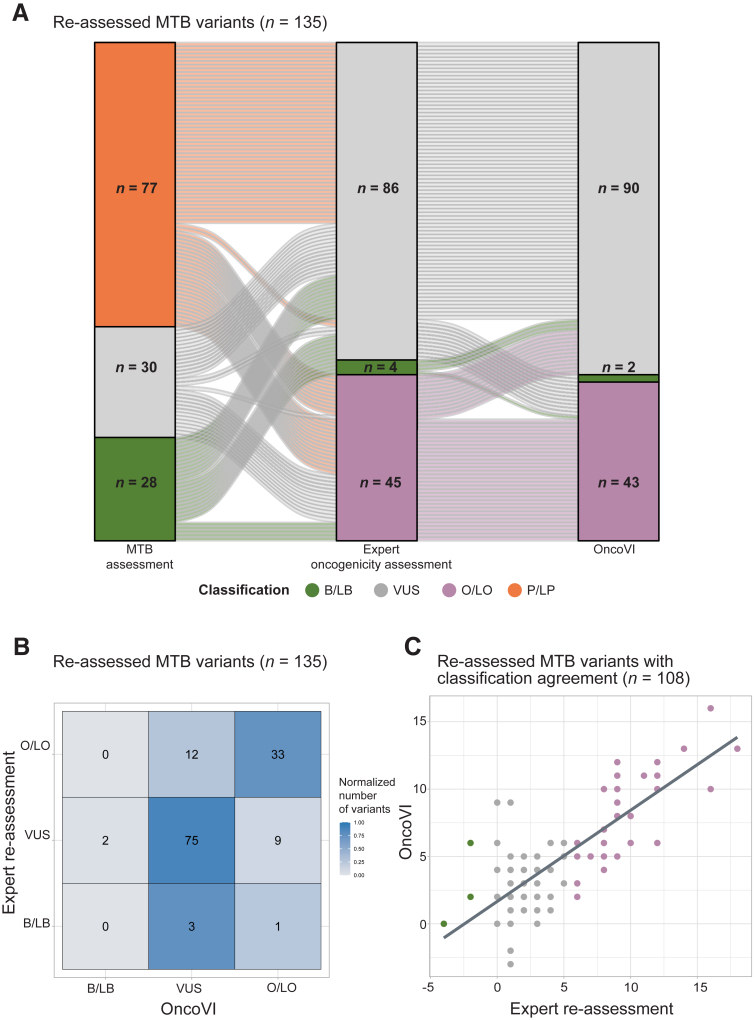
Results of OncoVI on the subset of Molecular Tumor Board (MTB) variants re-assessed by experts based on the oncogenicity guidelines. **A:** Alluvial plot of the 135 variants of the validation data set re-assessed by experts according to the oncogenicity guidelines. Horizontally distributed columns (axes) represent variants classified by the MTB (**left**), by the re-assessment of the experts (**middle**), and by OncoVI (**right**). Alluvial flows between axes show the correspondence between variants' classifications. **B:** Confusion matrix of the agreement between OncoVI and the expert re-assessment, based on the oncogenicity guidelines, in classifying the variants of the validation data set. The color scale indicates the normalized number of variants [ie, the ratio (calculated by row) between the number of variants of each cell and the total number of variants]. **C:** Scatterplot between the points assigned by the experts and the points assigned by OncoVI for the 108 variants with classification agreement. B/LB, benign/likely benign; O/LO, oncogenic/likely oncogenic; P/LP, pathogenic/likely pathogenic; VUS, variant of uncertain significance.

To better investigate the discrepancies between OncoVI and the MTB assessments, the expert cancer biologists re-evaluated a subset of 135 MTB variants (“validation data set”) using the oncogenicity guidelines and the resources used by OncoVI, while remaining blinded to OncoVI implementation of the criteria. This subset was selected on the basis of the comparison between OncoVI and MTB assessments (Figure 3A). Specifically, the 135 variants of the validation data set included the two variants classified within the MTB as P/LP and classified by OncoVI as B/LB and the 13 variants classified within the MTB as B/LB and classified by OncoVI as O/LO. Furthermore, randomly chosen variants classified as O/LO or VUS by OncoVI, both with and without agreement with the MTB assessment, were included (Supplemental Figure S5A and Supplemental Table S2). These 135 variants involved 104 different genes (67 OGs, 61 TSGs, and 7 with an unknown role), and were mainly truncating (*n* = 63), missense (*n* = 50), or splice site variants. Experts' classification based on the oncogenicity guidelines had an agreement of 34% (*n* = 46/135 variants) with the earlier MTB assessment (Figure 4A and Supplemental Figure S5B).

Although OncoVI agreement with the previous MTB assessment was 22% (*n* = 30/135) (Supplemental Figure S5A), the agreement with experts' re-assessment reached 80% (*n* = 108/135) (Figure 4B). No variants re-assessed by experts as O/LO had been classified as B/LB by OncoVI. In the 108 variants with classification agreement, the variant-specific scores assigned by experts and those by OncoVI showed a strong positive correlation (*r* = 0.7, *P* < 2.2 × 10^–^^16^) (Figure 4C). For the 33 O/LO variants, OncoVI had assigned the same or lower variant-specific scores (Supplemental Figure S6A and Supplemental Table S2), whereas for the 75 VUS variants, the scores by OncoVI were the same or higher (Supplemental Figure S7A and Supplemental Table S2). In addition, as in the SOP data set, the evaluation of the most frequently triggered criteria confirmed that OncoVI reliably activated strong oncogenic criteria, critical for accurate O/LO classification (Supplemental Figure S6B and Supplemental Figure S7B).

The analysis of all the criteria triggered by experts and OncoVI in the 108 variants with classification agreement (Supplemental Figure S8) revealed that the concordance was highest for OP4 (*n* = 107/108), OVS1 (*n* = 26/108), OS1 (*n* = 7/108), and OM2 (*n* = 8/108). Notably, for nine variants previously classified as VUS within MTB and reclassified as O/LO by experts and OncoVI, both methods triggered OVS1 (very strong, 8 points). A good concordance of the criteria triggered by both methods was exemplified by two MTB VUS variants in *PIK3CA* that were concordantly reclassified as O/LO (Table 2). Specifically, both variants were present in curated resources, such as CGI and ClinVar, on the basis of which both experts and OncoVI triggered the strong oncogenic criteria OS1 and OS2.

**Table 2:** Selected Variants of the Validation Data Set with Discrepancies between the MTB Assessment and OncoVI Oncogenicity Classification

Variant	MTB	Experts	OncoVI	Criteria (OncoVI)	Criteria (experts)
*IDH2*:p.Arg172Thr	P/LP	O/LO	VUS	OM1, OP1, OP3, OP4	OS2, OM1, OP1, OP3, OP4
*PIK3CA*:p.Arg93Gln	VUS	O/LO	O/LO	OS1, OS2, OM4, OP1, OP4	OS1, OS2, OM4, OP1, OP4
*PIK3CA*:p.Asn345Lys	VUS	O/LO	O/LO	OS1, OS2, OP1, OP4	OS1, OS2, OM3, OP1, OP4

MTB, Molecular Tumor Board; O/LO, oncogenic/likely oncogenic; OM1, oncogenic moderate-1 (2 points); OM3, oncogenic moderate-3 (2 points); OM4, oncogenic moderate-4 (2 points); OP1, oncogenic supporting-1 (1 point); OP3, oncogenic supporting-3 (1 point); OP4, oncogenic supporting-4 (1 point); OS1, oncogenic strong-1 (4 points); OS2, oncogenic strong-2 (4 points); P/LP, pathogenic/likely pathogenic; VUS, variant of uncertain significance.

OS2 (strong, 4 points) and SBS2 (strong, –4 points) were more frequently applied by experts alone, likely reflecting their ability to resolve variants with conflicting interpretation in ClinVar, compared with the automated approach. In addition, experts showed greater flexibility in triggering these criteria. For example, the MTB P/LP variant *IDH2*:p.Arg172Thr was classified by OncoVI as VUS with 5 points [ie, close to the threshold to LO, and confirmed by expert as O/LO as they additionally triggered the criterion OS2 (4 points) even if the annotation in ClinVar was without assertion criteria] (Table 2). Criteria with largest disagreement included OM1, OP1, and SBP1, all of moderate or supporting evidence. Overall, the adoption of the oncogenicity guidelines showed high agreement between experts and OncoVI with respect to the MTB classification based on variant effect on protein function, as reflected by the comparable variant-specific scores. In addition, OncoVI and experts strongly agreed on criteria requiring minimal interpretation of the resources (eg, OP4, OVS1, OS1, OS3, OM2, and OP3). Furthermore, discrepancies in OS2, OM1, OP1, SBS2, and SBP1 were likely because of experts' evaluation of the resources.

### Performance of OncoVI on the ClinVar Data Set

The ClinVar database has recently started providing oncogenicity classifications manually curated by the National Center for Biotechnology Information. As of April 2025, 691 variants (11%; *n* = 691/6113) (*https://ftp.ncbi.nlm.nih.gov/pub/clinvar/tab_delimited*, last accessed April 12, 2025) involving 227 genes were classified as benign: 1, likely benign: 1, likely oncogenic: 373, oncogenic: 58, or uncertain significance: 258 (ClinVar data set) (Supplemental Table S8). According to the COSMIC Cancer Gene Census, of these 691 variants, 343 occurred in TSGs, 163 occurred in OGs, and 111 had a dual role. The remaining 74 variants were located in genes with an unknown role. Functional annotation using the in-house VEP-based pipeline revealed that most variants were truncating (*n* = 313), including nonsense, frameshift, and start-loss variants, followed by missense variants (*n* = 280). Splice site–related variants accounted for 50 alterations, whereas in-frame insertions/deletions were 37; the remaining variants (*n* = 11) were synonymous, untranslated region, upstream, or noncoding. The three most frequently annotated genes were *ATRX* (*n* = 31), *TP53* (*n* = 28), and *EGFR* (*n* = 23). When considering ClinVar assessment as ground truth, OncoVI correctly classified 83% of the variants (*n* = 573/691) with a sensitivity of 84% (*n* = 362/431) for the O/LO class. No O/LO variants were misclassified as B/LB by OncoVI or vice versa.

In the 69 O/LO variants classified by OncoVI as VUS, OncoVI mainly triggered supporting (1 point) or moderate (2 points) oncogenic criteria (OP4: *n* = 69/69; OP1: *n* = 48/69; and OM1: *n* = 35/69). In addition, 55% (*n* = 38/69) of the variants had variant-specific scores of four and five (ie, close to the upper score limit of the VUS class). Furthermore, the majority (*n* = 32/48) of the 48 variants classified by ClinVar as VUS and reclassified by OncoVI as O/LO had variant-specific scores of 9 and 10 (ie, near the cutoff between the LO and O classes), most likely because of the triggered OVS1 (8 points) for null variants in a bona fide TSG. When evaluating the performance stratified by gene classes (OGs versus TSGs), higher concordance for variants in TSGs compared with OGs was observed (85.7% versus 76.1%) (Supplemental Table S6), in line with the SOP data set. In contrast, higher concordance for truncating alterations compared with missense was observed (87.5% versus 81.1%) (Supplemental Table S6).

Taken together, in addition to the good performance of OncoVI in the SOP data set and the MTB data set, OncoVI showed good agreement also with the oncogenicity classifications in ClinVar (Supplemental Figure S9).

## Discussion

The precise and reproducible classification of the oncogenicity of somatic variants is challenging in precision oncology. OncoVI can facilitate the broader adoption of the oncogenicity guidelines, promoting standardized oncogenicity classification. Its implementation required defining the biological meaning of each criterion and selecting the most appropriate publicly available resources, with expert consensus.

Testing OncoVI on the SOP data set revealed high agreement with the SOP oncogenicity classification, with variant-specific scores strongly correlating with those manually assigned by the SOP. Although OncoVI scores were in general higher, this is in line with the intention to minimize missed real oncogenic variants at the cost of potential false positives for expert review. The analysis of the triggered criteria confirmed their correct interpretation and the appropriate use of resources in OncoVI. Notably, OS1 and OS2 were the strongest associated criteria with a correct oncogenic classification. Minor differences with the SOP arose at the level of individual triggered criteria, likely because of OncoVI use of an external data set of oncogenic variants for OS1 and OM3, which required a set of “previously established oncogenic variants.” according to the oncogenicity guidelines. Additionally, OS3 was rarely triggered by OncoVI because, according to the guidelines, OS3 is not applicable if OS1 has been activated. Apart from these factors that likely contributed to OncoVI higher variant-specific scores, its evaluation closely aligned with the SOP manual application of the criteria, with the advantage of an automated evaluation.

The real-world MTB data set had greater gene heterogeneity than the SOP data set and a higher proportion of missense variants. Despite this, OncoVI showed good concordance with the MTB assessment based on variant effect on protein function. In particular, a higher concordance between MTB and OncoVI was observed for missense variants. This suggests that more information on these variants may be available in the resources used by OncoVI. On the other hand, this also highlights the need for expert review in the interpretation of complex truncating variants. As for the SOP data set, the agreement between OncoVI O/LO variants and MTB P/LP variants was higher than for B/LB variants. Two MTB P/LP variants classified by OncoVI as B/LB, likely because of changing ClinVar evidence, were re-assessed by the experts as VUS, highlighting the value of automated systems that ensure reproducibility through consistent use of current data.^39^ Conversely, the 13 B/LB MTB variants classified by OncoVI as O/LO outlined OncoVI limitations in solving conflicting classifications of pathogenicity in ClinVar, which could be mitigated by integrating medical literature text mining tools, like CancerMine.^40^ Challenges also arose in the correct classification of intronic variants or truncating variants in the terminal exons, which often do not affect protein function (eg, stop codons in the last exon that are unlikely to activate nonsense-mediated mRNA decay^41^). These factors may also explain the differences identified in the MTB VUS variants classified as O/LO by OncoVI.

Most of the discrepancies between MTB and OncoVI classifications reflected their conceptual differences (ie, the fact that MTB assessed variant effect on protein function, whereas OncoVI focused on oncogenicity). Many truncating and splice site variants were deemed P/LP in the MTB but classified as VUS by OncoVI, likely because of their localization outside bona fide TSGs that precluded the activation of OVS1 (very strong, 8 points) by OncoVI. These findings suggest that the guidelines might be refined by either allowing null variants in non–bona fide TSGs to trigger OVS1 with a reduced score (eg, 4 instead of 8); or including new criteria for clinically relevant gain-of-function variants in OGs.

The higher proportion of variants in the MTB data set triggering OVS1, compared with the SOP data set, may reflect the higher prevalence of variants in TSGs in this real-world data set. In addition, the less frequent activation of criteria dependent on curated databases (eg, OS1) likely suggests that such databases, covering mainly well-characterized variants, may be insufficient for interpreting rare variants. In contrast, the comparable frequency of SBS2 activation across both data sets (SOP and MTB) may stem from the predominance of B/LB variants in ClinVar (*n* = 1,196,965 benign/likely benign versus *n* = 131,059 pathogenic/likely pathogenic; *https://ftp.ncbi.nlm.nih.gov/pub/clinvar/tab_delimited*, last accessed October 14, 2024).

Experts' re-assessment of the validation data set using the oncogenicity guidelines elucidated the discrepancies between MTB and OncoVI classifications. The variants classified as VUS within the MTB and as O/LO by OncoVI were often confirmed by the experts, likely because of the activation of OVS1 (very strong, 8 points, “Null variants in a bona fide TSG”). These variants were predominantly truncating in the far C-terminal protein region, which may allow residual protein function and explain why the experts had previously classified these variants as VUS within the MTB. Reducing OVS1-associated points for these variants may solve the misclassification. On the other hand, some exemplary MTB VUS variants reclassified as O/LO by both experts and OncoVI underline the value of standardized oncogenicity guidelines in reducing uncertainty in variant interpretation and supporting variant prioritization in clinically relevant genes. Overall, the experts' re-assessment confirmed OncoVI accurate implementation of the guidelines. In addition, the agreement between OncoVI and experts was higher for criteria not requiring subjective interpretation of the resources than for criteria requiring experts' judgment (eg, involving the predictive scores from the computational algorithms used for OP1 and SBP1). This divergence likely reflects differences in the interpretation of online resources or in the thresholds applied by experts to define deleterious/benign predictions, while also highlighting the value of a reproducible, automated approach. In addition, expert reassessment of some MTB P/LP variants classified by OncoVI as VUS (5 points) demonstrated experts' greater flexibility in resource interpretation, whereas OncoVI applied the criteria more stringently. Together, these findings suggest that VUS variants with OncoVI score close to the LO threshold warrant careful expert evaluation.

Collectively, OncoVI is a valuable tool that supports a broader adoption of the oncogenicity guidelines for somatic variant classification. The adoption of OncoVI on further large, heterogeneous cohorts would also allow assessing the benefits of the above-discussed potential changes as well suggest additional optimizations to the rules and/or associated points. In addition, OncoVI is distributed as a Python-based open-source tool, allowing the integration and customization into pre-existing pipelines.

## Disclosure Statement

A.Ha. has received honoraria for lectures or consulting/advisory boards for AbbVie, Agilent, AstraZeneca, Biocartis, BMS, Boehringer Ingelheim, Cepheid, Diaceutics, Gilead, Illumina, Ipsen, Janssen, Lilly, Merck, MSD, Nanostring, Novartis, Pfizer, Qiagen, QUIP GmbH, Roche, Sanofi, and 3Dhistech; and other research support from AstraZeneca, Biocartis, Cepheid, Gilead, Illumina, Janssen, Nanostring, Novartis, Owkin, Qiagen, QUIP GmbH, Roche, and Sanofi. F.H. has received honoraria for lectures or consulting/advisory boards for AstraZeneca, BMS, Boehringer Ingelheim, and Novartis; and receives research support from Illumina GmbH.

## Supplemental Data

Supplemental Figure S1 Results on the variants of the standard operating procedure (SOP) data set correctly classified as oncogenic/likely oncogenic (O/LO). **A:** Dumbbell plot of the 38 variants correctly classified as O/LO. Horizontal bars indicate the classification of the variants according to the SOP point-based system (ie, score ≥10: oncogenic; 6 ≤ score ≤ 9: likely oncogenic). **B:** Bar plot of the criteria triggered by OncoVI in the 38 variants correctly classified as O/LO. Criteria are sorted according to decreasing corresponding points: OVS1, oncogenic very strong-1 (8 points); OS1, oncogenic strong-1 (4 points); OS2, oncogenic strong-2 (4 points); OS3, oncogenic strong-3 (4 points); OM1, oncogenic moderate-1 (2 points); OM2, oncogenic moderate-2 (2 points); OM3, oncogenic moderate-3 (2 points); OM4, oncogenic moderate-4 (2 points); OP1, oncogenic supporting-1 (1 point); OP3, oncogenic supporting-3 (1 point); OP4, oncogenic supporting-4 (1 point); SBP1, somatic benign supporting-1 (–1 point).

Supplemental Figure S2 Results on the benign/likely benign (B/LB) and variant of uncertain significance (VUS) variants of the standard operating procedure (SOP) data set with classification agreement between the SOP and OncoVI. **A:** Dumbbell plot of the 10 variants correctly classified as B/LB. Horizontal bars indicate the classification of the variants according to the SOP point-based system (ie, –6 ≤ score ≤ –1: likely benign; score ≤ –7: benign). **B:** Bar plot of the criteria triggered by OncoVI in the 10 variants correctly classified as B/LB, sorted according to decreasing corresponding points. **C:** Dumbbell plot of the 27 SOP VUSs with classification agreement between the SOP and OncoVI. Horizontal bar indicates the classification of the variants according to the SOP point-based system (ie, 0 ≤ score ≤ 5: VUS). **D:** Bar plot of the criteria triggered by OncoVI in the 27 SOP VUS variants with classification agreement. Criteria are sorted according to decreasing corresponding points: OS1, oncogenic strong-1 (4 points); OS3, oncogenic strong-3 (4 points); OM1, oncogenic moderate-1 (2 points); OP1, oncogenic supporting-1 (1 point); OP3, oncogenic supporting-3 (1 point); OP4, oncogenic supporting-4 (1 point); SBP1, somatic benign supporting-1 (–1 point); SBS2, somatic benign strong-2 (–4 points); SBS1, somatic benign strong-1 (–4 points); SBVS1, somatic benign very strong-1 (–8 points).

Supplemental Figure S3 Molecular Tumor Board (MTB) data set of real-world routine diagnostic variants. **A:** Distribution of the 557 patients of the MTB cohort across tumor entities. **B:** Distribution of the 7802 variants of the MTB data set across the MTB assessment classes. CNS, central nervous system; CUP, cancer of unknown primary; Lung, NSCLC, non–small-cell lung cancer; VUS, variant of uncertain significance.

Supplemental Figure S4 Criteria triggered for the benign/likely benign (B/LB) and variant of uncertain significance (VUS) variants of the Molecular Tumor Board (MTB) data set with classification agreement. **A:** Bar plot of the criteria triggered by OncoVI in the 961 variants classified as B/LB by both MTB and OncoVI. Criteria are sorted according to decreasing corresponding points. **B:** Bar plot of the criteria triggered by OncoVI in the 3955 variants classified as VUS by both MTB and OncoVI. Criteria are sorted according to decreasing corresponding points: OVS1, oncogenic very strong-1 (8 points); OS1, oncogenic strong-1 (4 points); OS3, oncogenic strong-3 (4 points); OM1, oncogenic moderate-1 (2 points); OM2, oncogenic moderate-2 (2 points); OM3, oncogenic moderate-3 (2 points); OM4, oncogenic moderate-4 (2 points); OP1, oncogenic supporting-1 (1 point); OP3, oncogenic supporting-3 (1 point); OP4, oncogenic supporting-4 (1 point); SBP1, somatic benign supporting-1 (–1 point); SBS2, somatic benign strong-2 (–4 points); SBS1, somatic benign strong-1 (–4 points); SBVS1, somatic benign very strong-1 (–8 points).

Supplemental Figure S5 Molecular Tumor Board (MTB) variants of the validation data set. **A:** Confusion matrix of the agreement between OncoVI oncogenicity and MTB assessment in classifying the 135 variants of the validation data set. **B:** Confusion matrix of the agreement between MTB and expert re-assessment, based on the oncogenicity guidelines, in classifying the 135 variants of the validation data set. The color scale indicates the normalized number of variants [ie, the ratio (calculated by row) between the number of variants of each cell and the total number of variants). B/LB, benign/likely benign; O/LO, oncogenic/likely oncogenic; P/LP, pathogenic/likely pathogenic; VUS, variant of uncertain significance.

Supplemental Figure S6 Results on the variants re-assessed as oncogenic/likely oncogenic (O/LO) with agreement between expert and OncoVI classification. **A:** Dumbbell plot of the 33 variants classified as O/LO by both experts and OncoVI. Horizontal bars indicate the classification of the variants according to the standard operating procedure point-based system (ie, score ≥ 10: oncogenic; 6 ≤ score ≤ 9: likely oncogenic). **B:** Bar plot of the criteria triggered by OncoVI in the 33 variants classified as O/LO by both experts and OncoVI. Criteria are sorted according to decreasing corresponding points: OVS1, oncogenic very strong-1 (8 points); OS1, oncogenic strong-1 (4 points); OS2, oncogenic strong-2 (4 points); OM1, oncogenic moderate-1 (2 points); OM4, oncogenic moderate-4 (2 points); OP1, oncogenic supporting-1 (1 point); OP4, oncogenic supporting-4 (1 point); SBP1, somatic benign supporting-1 (–1 point).

Supplemental Figure S7 Results on the variants re-assessed as variant of uncertain significance (VUS) with agreement between expert and OncoVI classification. **A:** Dumbbell plot of the 75 variants classified as VUS by both experts and OncoVI. Horizontal bars indicate the classification of the variants according to the standard operating procedure point-based system (ie, 0 ≤ score ≤ 5: VUS). **B:** Bar plot of the criteria triggered by OncoVI in the 75 variants classified as VUS by both experts and OncoVI. Criteria are sorted according to decreasing corresponding points: OVS1, oncogenic very strong-1 (8 points); OS2, oncogenic strong-2 (4 points); OM1, oncogenic moderate-1 (2 points); OM2, oncogenic moderate-2 (2 points); OM4, oncogenic moderate-4 (2 points); OP1, oncogenic supporting-1 (1 point); OP3, oncogenic supporting-3 (1 point); OP4, oncogenic supporting-4 (1 point); SBP1, somatic benign supporting-1 (–1 point); SBS2, somatic benign strong-2 (–4 points).

Supplemental Figure S8 Re-assessed Molecular Tumor Board (MTB) variants with agreement between expert and OncoVI classification. Each row corresponds to 1 of the 108 variants with agreement between expert and OncoVI classification, and each column corresponds to an assessed criterion. The color of the cell indicates whether a criterion was triggered by the experts only (dark gold), OncoVI only (red), both (light blue), or none of the two (gray). The bar plot on the left indicates the classification of each variant according to the MTB, OncoVI, and expert assessment. B/LB, benign/likely benign; O/LO, oncogenic/likely oncogenic; OM1, oncogenic moderate-1 (2 points); OM2, oncogenic moderate-2 (2 points); OM3, oncogenic moderate-3 (2 points); OM4, oncogenic moderate-4 (2 points); OP1, oncogenic supporting-1 (1 point); OP3, oncogenic supporting-3 (1 point); OP4, oncogenic supporting-4 (1 point); OS1, oncogenic strong-1 (4 points); OS2, oncogenic strong-2 (4 points); OS3, oncogenic strong-3 (4 points); OVS1, oncogenic very strong-1 (8 points); P/LP, pathogenic/likely pathogenic; SBS1, somatic benign strong-1 (–4 points); SBS2, somatic benign strong-2 (–4 points); SBP1, somatic benign supporting-1 (–1 point); SBP2, somatic benign supporting-2 (–1 point); SBVS1, somatic benign very strong-1 (–8 points); VUS, variant of uncertain significance.

Supplemental Figure S9 OncoVI concordance with reference classification. Schematic summary of the three data sets used to evaluate OncoVI performance: the standard operating procedure (SOP) data set, the real-world Molecular Tumor Board (MTB) data set, and the ClinVar data set. For each data set, the number of analyzed variants and the concordance between OncoVI oncogenicity classification and the corresponding reference classification are shown.
