# Supplementary material for: Oncogenicity Variant Interpreter (OncoVI) Supports Harmonized Somatic Variant Interpretation in Precision Oncology
Source: J Mol Diagn. 2026 Apr 3;28(6):469–84. doi: 10.1016/j.jmoldx.2026.03.004 (PMC13269341; doi:10.1016/j.jmoldx.2026.03.004)

Supp.Figure 1

A

Scores of the SOP variants correctly classified as O/LO (n=38)

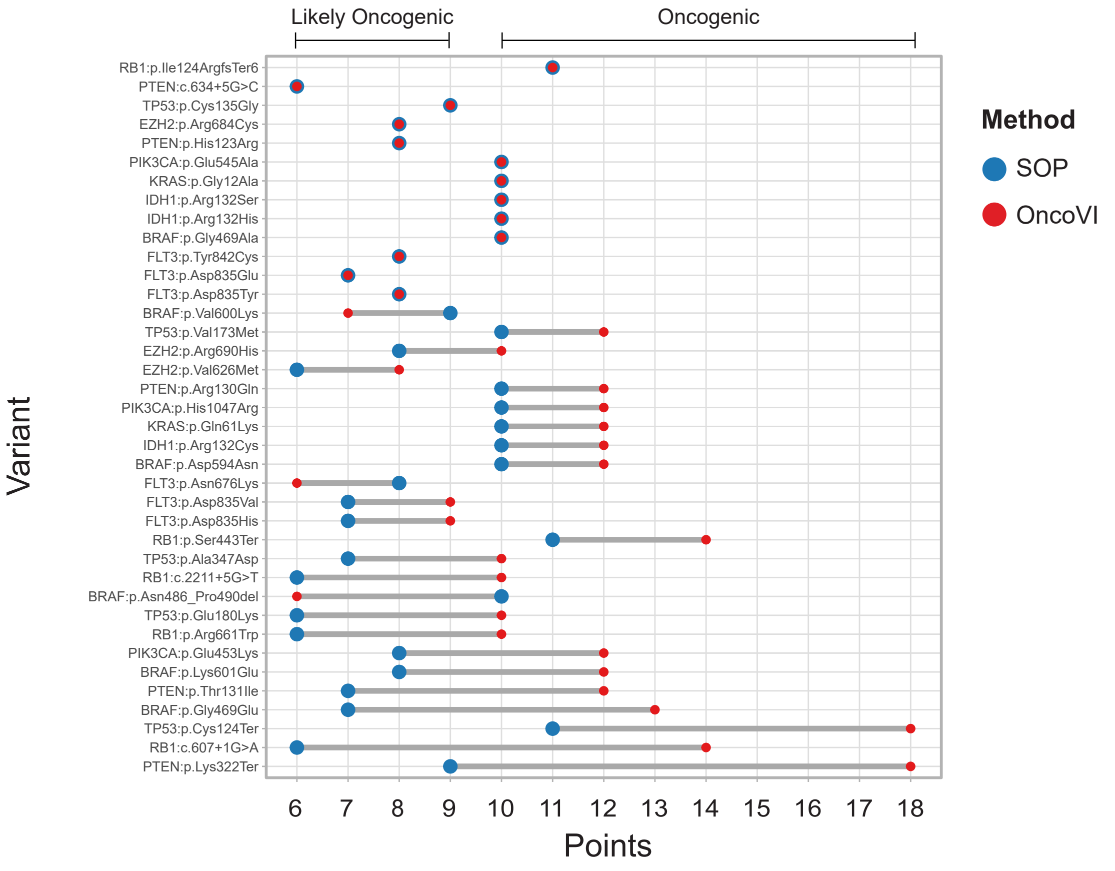

B

Criteria triggered by OncoVI for the SOP variants correctly classified as O/LO (n=38)

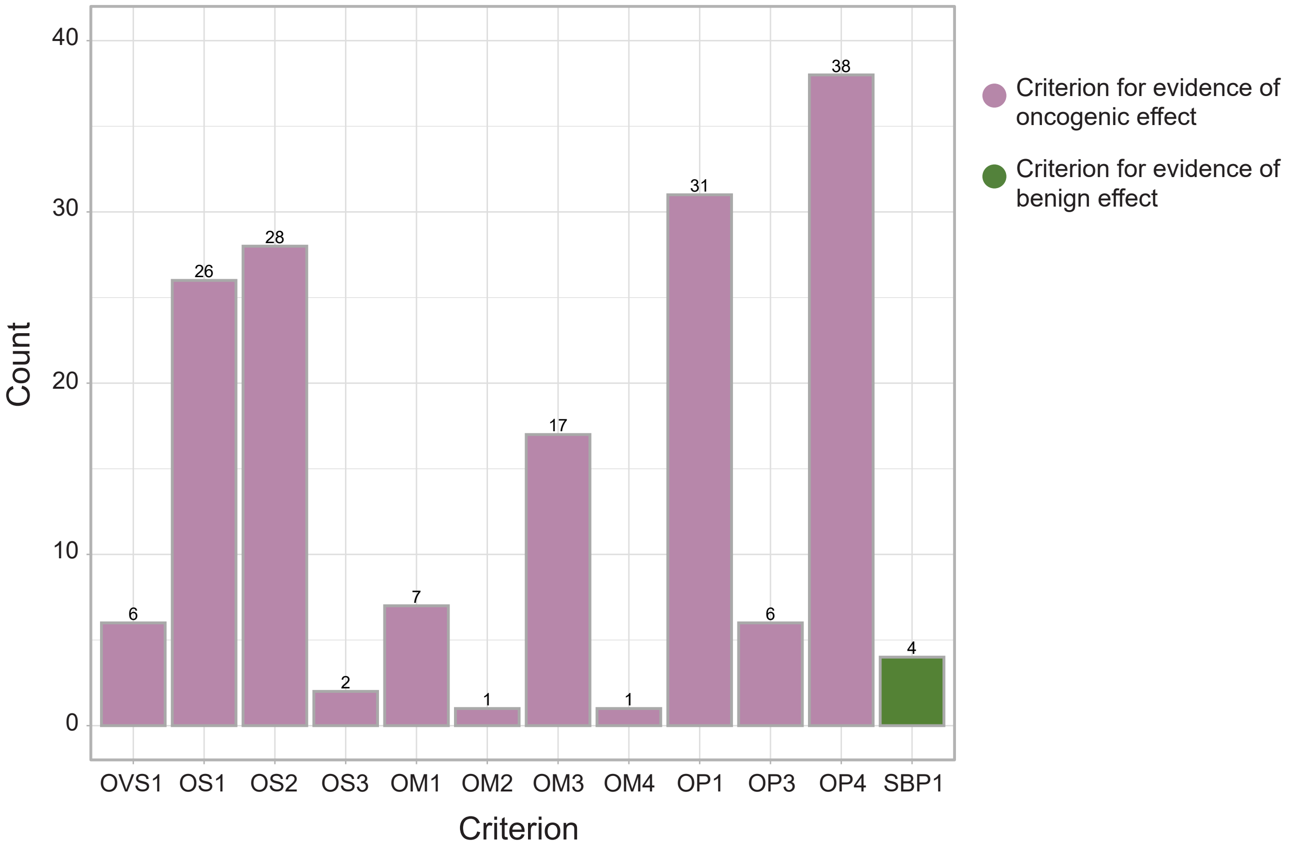

Supplement: Supplemental Figure S1 — Results on the variants of the standard operating procedure (SOP) data set correctly classified as oncogenic/likely oncogenic (O/LO). A: Dumbbell plot of the 38 variants correctly classified as O/LO. Horizontal bars indicate the classification of the variants according to the SOP point-based system (ie, score ≥10: oncogenic; 6 ≤ score ≤ 9: likely oncogenic). B: Bar plot of the criteria triggered by OncoVI in the 38 variants correctly classified as O/LO. Criteria are sorted according to decreasing corresponding points: OVS1, oncogenic very strong-1 (8 points); OS1, oncogenic strong-1 (4 points); OS2, oncogenic strong-2 (4 points); OS3, oncogenic strong-3 (4 points); OM1, oncogenic moderate-1 (2 points); OM2, oncogenic moderate-2 (2 points); OM3, oncogenic moderate-3 (2 points); OM4, oncogenic moderate-4 (2 points); OP1, oncogenic supporting-1 (1 point); OP3, oncogenic supporting-3 (1 point); OP4, oncogenic supporting-4 (1 point); SBP1, somatic benign supporting-1 (–1 point). [file mmc1.pdf]
