# Supplementary material for: Oncogenicity Variant Interpreter (OncoVI) Supports Harmonized Somatic Variant Interpretation in Precision Oncology
Source: J Mol Diagn. 2026 Apr 3;28(6):469–84. doi: 10.1016/j.jmoldx.2026.03.004 (PMC13269341; doi:10.1016/j.jmoldx.2026.03.004)

Supp.Figure 2

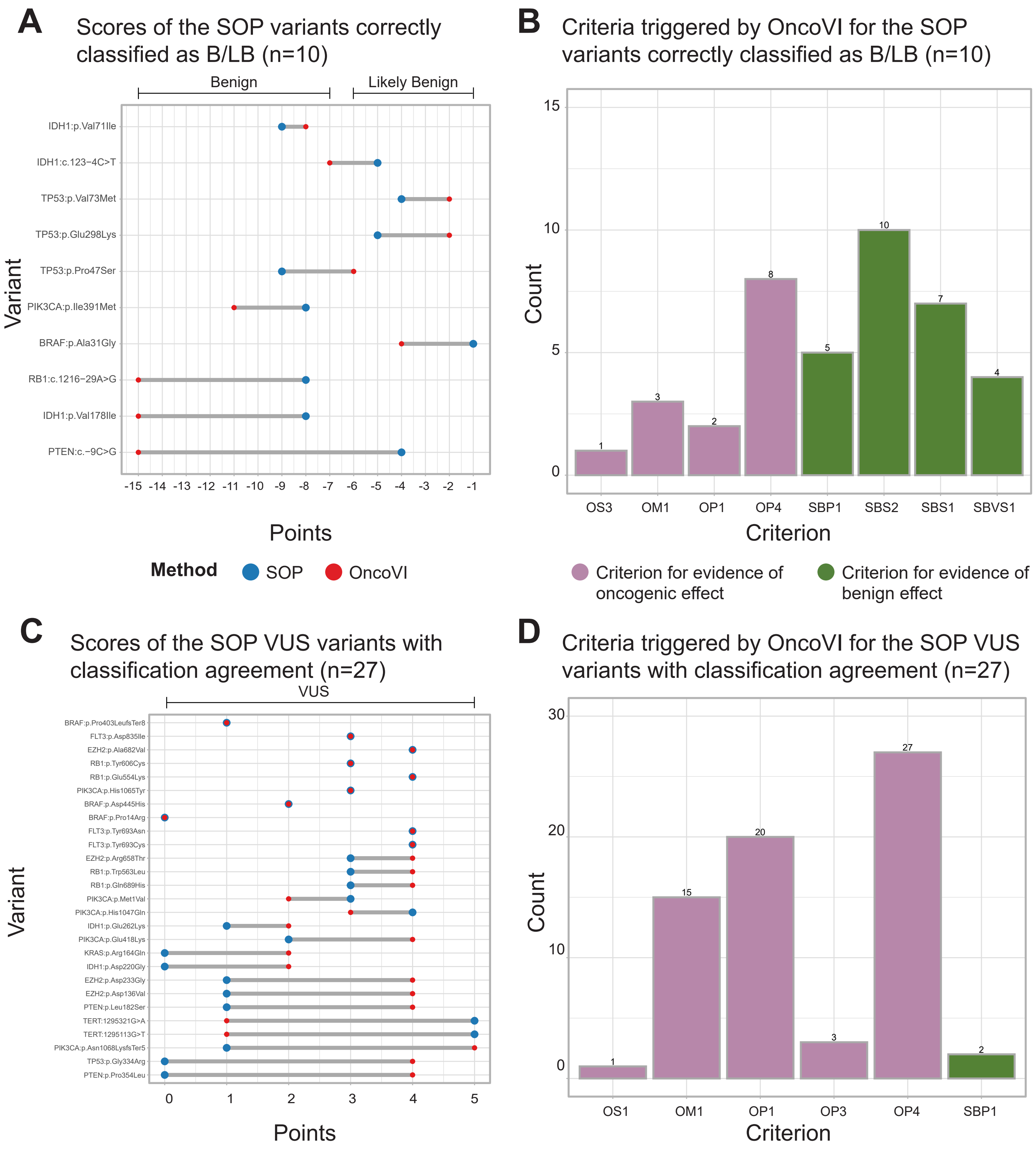

Supplement: Supplemental Figure S2 — Results on the benign/likely benign (B/LB) and variant of uncertain significance (VUS) variants of the standard operating procedure (SOP) data set with classification agreement between the SOP and OncoVI. A: Dumbbell plot of the 10 variants correctly classified as B/LB. Horizontal bars indicate the classification of the variants according to the SOP point-based system (ie, –6 ≤ score ≤ –1: likely benign; score ≤ –7: benign). B: Bar plot of the criteria triggered by OncoVI in the 10 variants correctly classified as B/LB, sorted according to decreasing corresponding points. C: Dumbbell plot of the 27 SOP VUSs with classification agreement between the SOP and OncoVI. Horizontal bar indicates the classification of the variants according to the SOP point-based system (ie, 0 ≤ score ≤ 5: VUS). D: Bar plot of the criteria triggered by OncoVI in the 27 SOP VUS variants with classification agreement. Criteria are sorted according to decreasing corresponding points: OS1, oncogenic strong-1 (4 points); OS3, oncogenic strong-3 (4 points); OM1, oncogenic moderate-1 (2 points); OP1, oncogenic supporting-1 (1 point); OP3, oncogenic supporting-3 (1 point); OP4, oncogenic supporting-4 (1 point); SBP1, somatic benign supporting-1 (–1 point); SBS2, somatic benign strong-2 (–4 points); SBS1, somatic benign strong-1 (–4 points); SBVS1, somatic benign very strong-1 (–8 points). [file mmc2.pdf]
