# Supplementary material for: Oncogenicity Variant Interpreter (OncoVI) Supports Harmonized Somatic Variant Interpretation in Precision Oncology
Source: J Mol Diagn. 2026 Apr 3;28(6):469–84. doi: 10.1016/j.jmoldx.2026.03.004 (PMC13269341; doi:10.1016/j.jmoldx.2026.03.004)

Supp.Figure 3

A

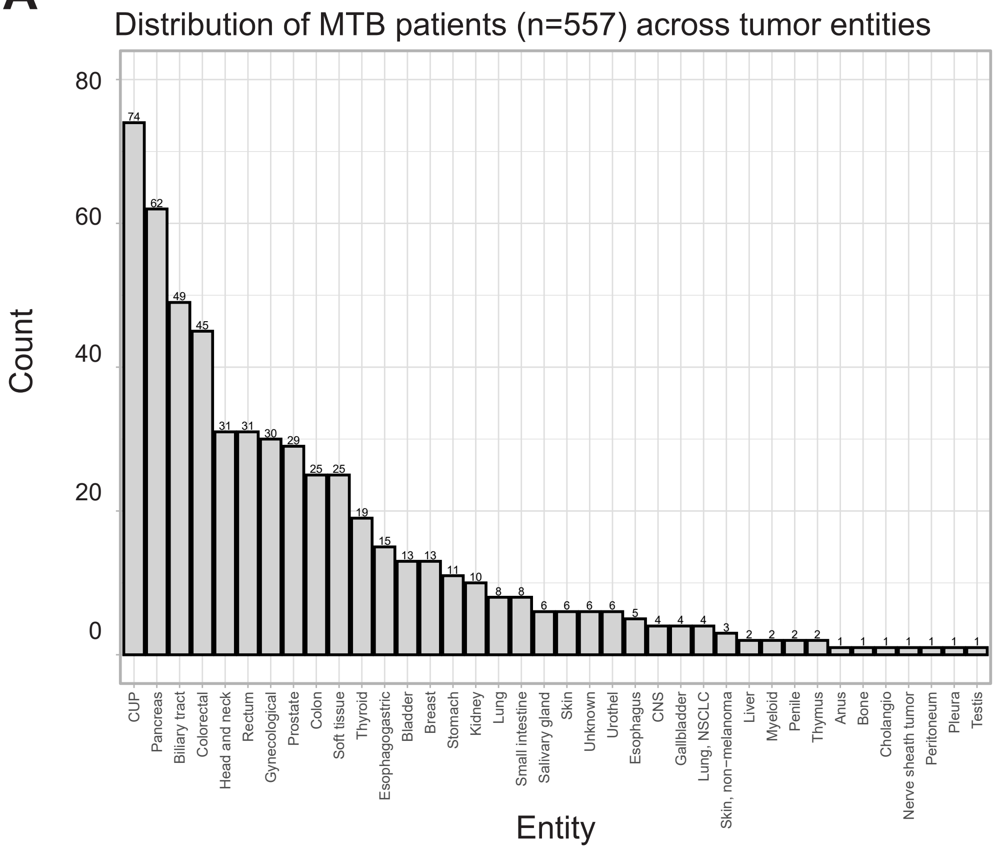

B

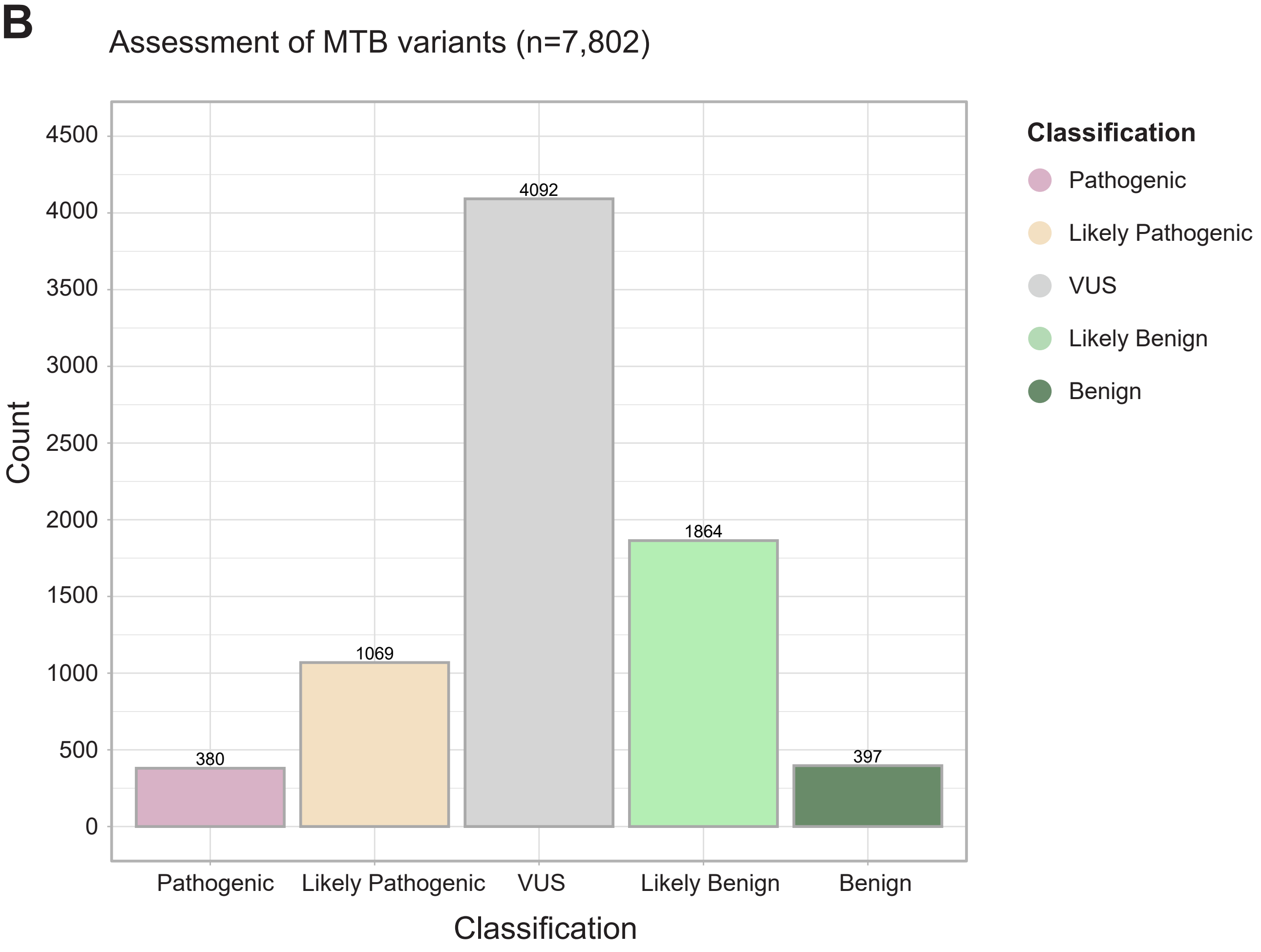

Supplement: Supplemental Figure S3 — Molecular Tumor Board (MTB) data set of real-world routine diagnostic variants. A: Distribution of the 557 patients of the MTB cohort across tumor entities. B: Distribution of the 7802 variants of the MTB data set across the MTB assessment classes. CNS, central nervous system; CUP, cancer of unknown primary; Lung, NSCLC, non–small-cell lung cancer; VUS, variant of uncertain significance. [file mmc3.pdf]
