# Supplementary material for: Oncogenicity Variant Interpreter (OncoVI) Supports Harmonized Somatic Variant Interpretation in Precision Oncology
Source: J Mol Diagn. 2026 Apr 3;28(6):469–84. doi: 10.1016/j.jmoldx.2026.03.004 (PMC13269341; doi:10.1016/j.jmoldx.2026.03.004)

Supp.Figure 4

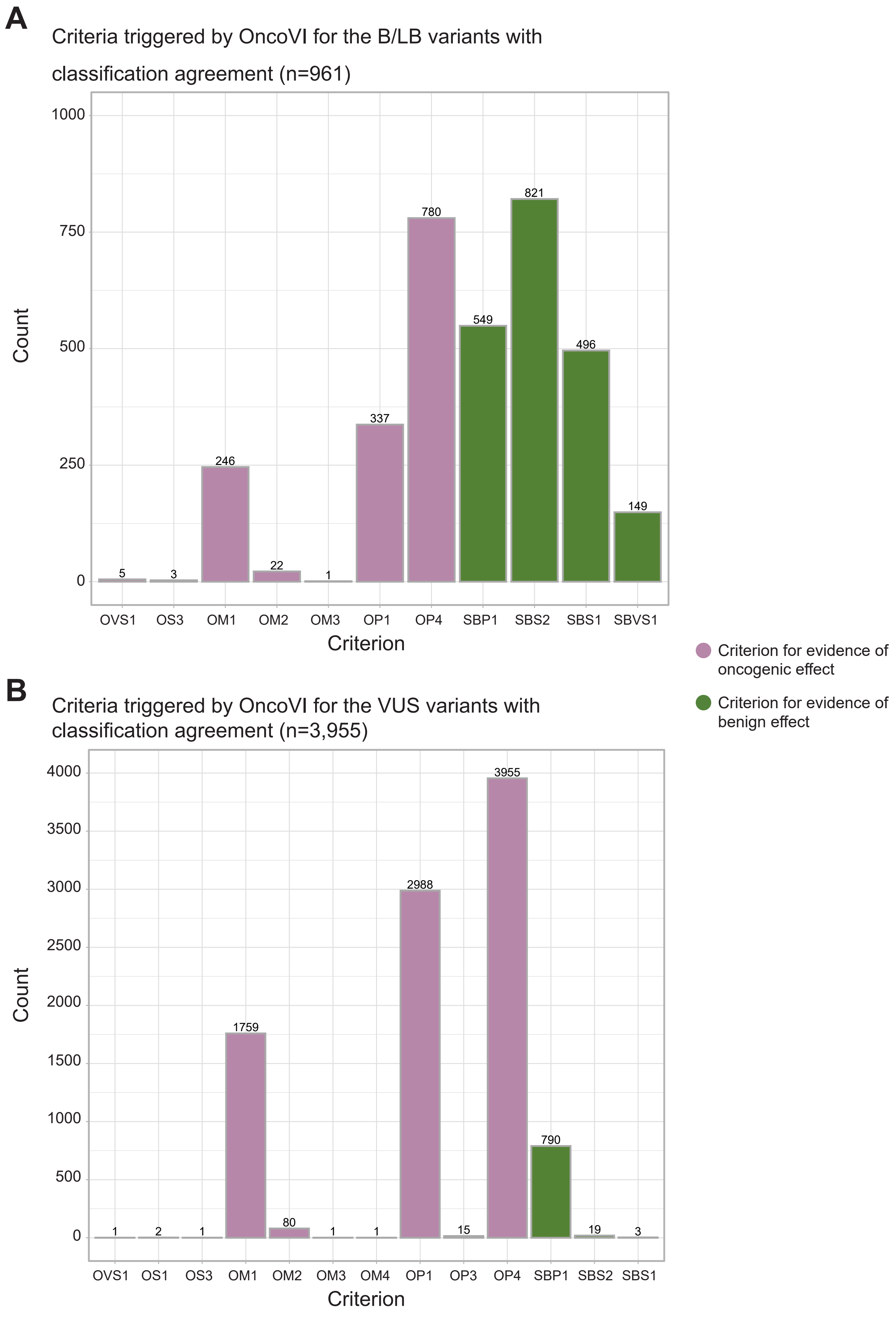

Supplement: Supplemental Figure S4 — Criteria triggered for the benign/likely benign (B/LB) and variant of uncertain significance (VUS) variants of the Molecular Tumor Board (MTB) data set with classification agreement. A: Bar plot of the criteria triggered by OncoVI in the 961 variants classified as B/LB by both MTB and OncoVI. Criteria are sorted according to decreasing corresponding points. B: Bar plot of the criteria triggered by OncoVI in the 3955 variants classified as VUS by both MTB and OncoVI. Criteria are sorted according to decreasing corresponding points: OVS1, oncogenic very strong-1 (8 points); OS1, oncogenic strong-1 (4 points); OS3, oncogenic strong-3 (4 points); OM1, oncogenic moderate-1 (2 points); OM2, oncogenic moderate-2 (2 points); OM3, oncogenic moderate-3 (2 points); OM4, oncogenic moderate-4 (2 points); OP1, oncogenic supporting-1 (1 point); OP3, oncogenic supporting-3 (1 point); OP4, oncogenic supporting-4 (1 point); SBP1, somatic benign supporting-1 (–1 point); SBS2, somatic benign strong-2 (–4 points); SBS1, somatic benign strong-1 (–4 points); SBVS1, somatic benign very strong-1 (–8 points). [file mmc4.pdf]
