# Supplementary material for: Oncogenicity Variant Interpreter (OncoVI) Supports Harmonized Somatic Variant Interpretation in Precision Oncology
Source: J Mol Diagn. 2026 Apr 3;28(6):469–84. doi: 10.1016/j.jmoldx.2026.03.004 (PMC13269341; doi:10.1016/j.jmoldx.2026.03.004)

Supp. Figure 5

A

Agreement between MTB and OncoVI on the MTB validation data set (n=135)

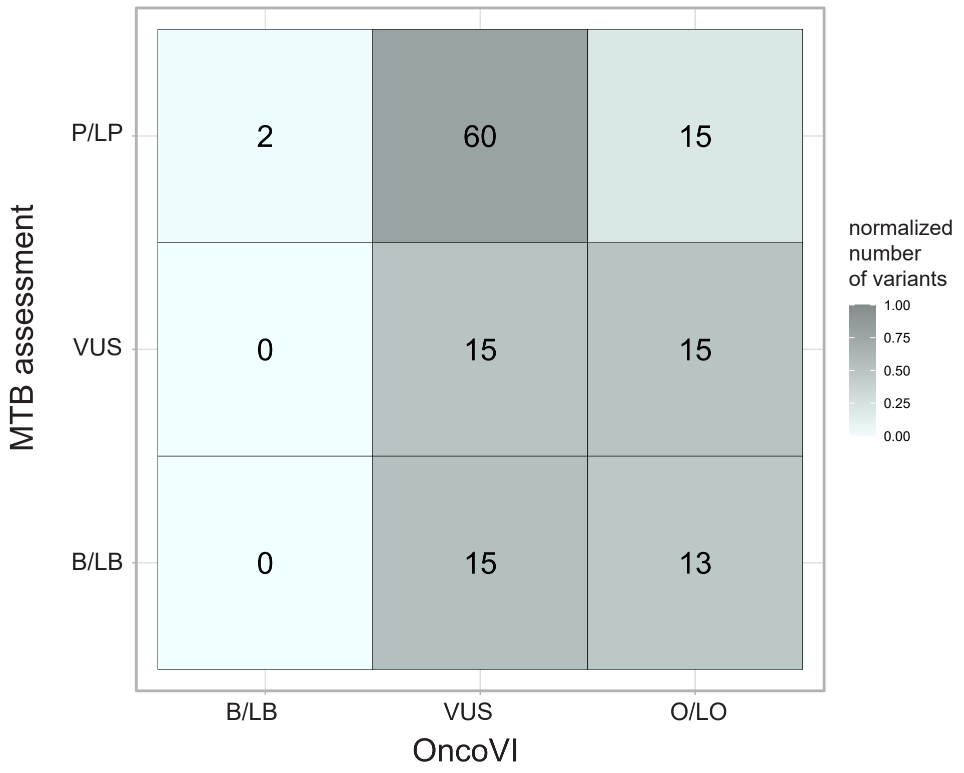

B

Agreement between MTB and experts on the MTB validation data set (n=135)

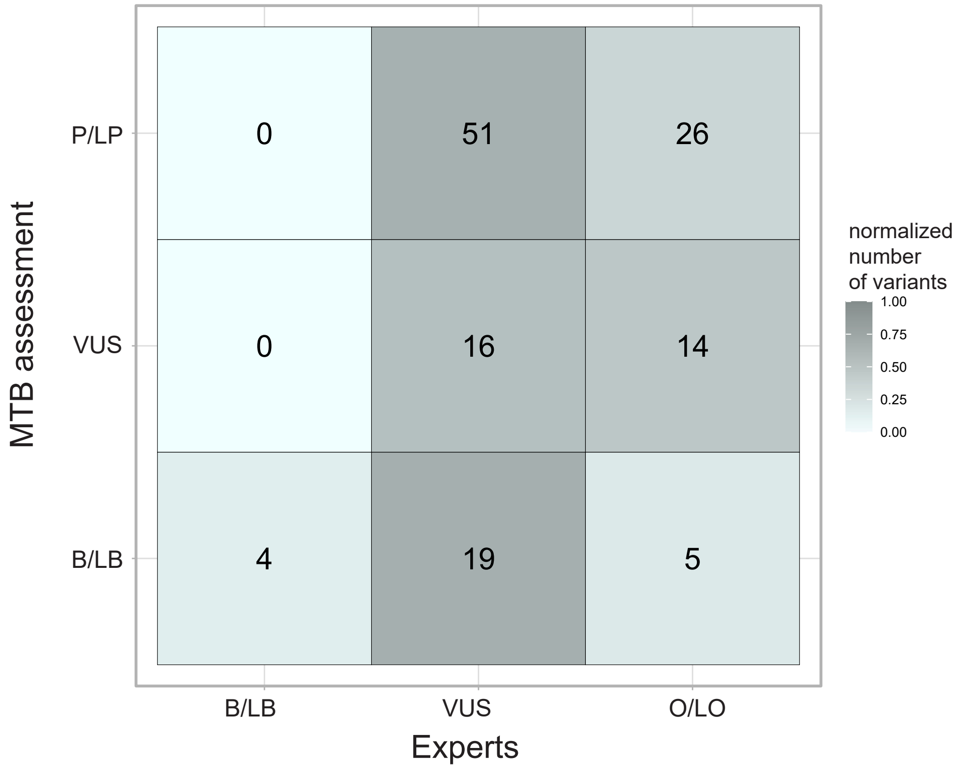

Supplement: Supplemental Figure S5 — Molecular Tumor Board (MTB) variants of the validation data set. A: Confusion matrix of the agreement between OncoVI oncogenicity and MTB assessment in classifying the 135 variants of the validation data set. B: Confusion matrix of the agreement between MTB and expert re-assessment, based on the oncogenicity guidelines, in classifying the 135 variants of the validation data set. The color scale indicates the normalized number of variants [ie, the ratio (calculated by row) between the number of variants of each cell and the total number of variants). B/LB, benign/likely benign; O/LO, oncogenic/likely oncogenic; P/LP, pathogenic/likely pathogenic; VUS, variant of uncertain significance. [file mmc5.pdf]
