# Supplementary material for: Oncogenicity Variant Interpreter (OncoVI) Supports Harmonized Somatic Variant Interpretation in Precision Oncology
Source: J Mol Diagn. 2026 Apr 3;28(6):469–84. doi: 10.1016/j.jmoldx.2026.03.004 (PMC13269341; doi:10.1016/j.jmoldx.2026.03.004)

Supp.Figure 6

A

Scores of the O/LO variants with agreement in OncoVI and expert classification (n=33)

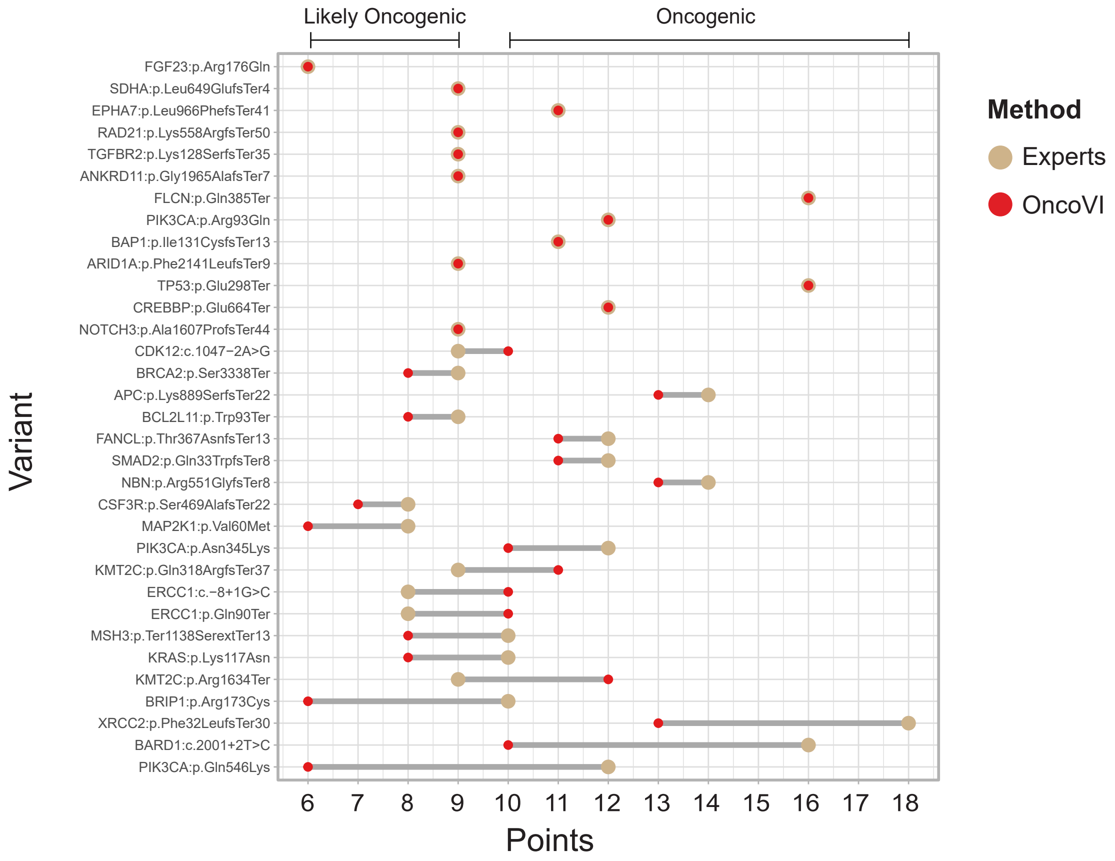

B

Criteria of the O/LO variants with agreement between OncoVI and expert classification (n=33)

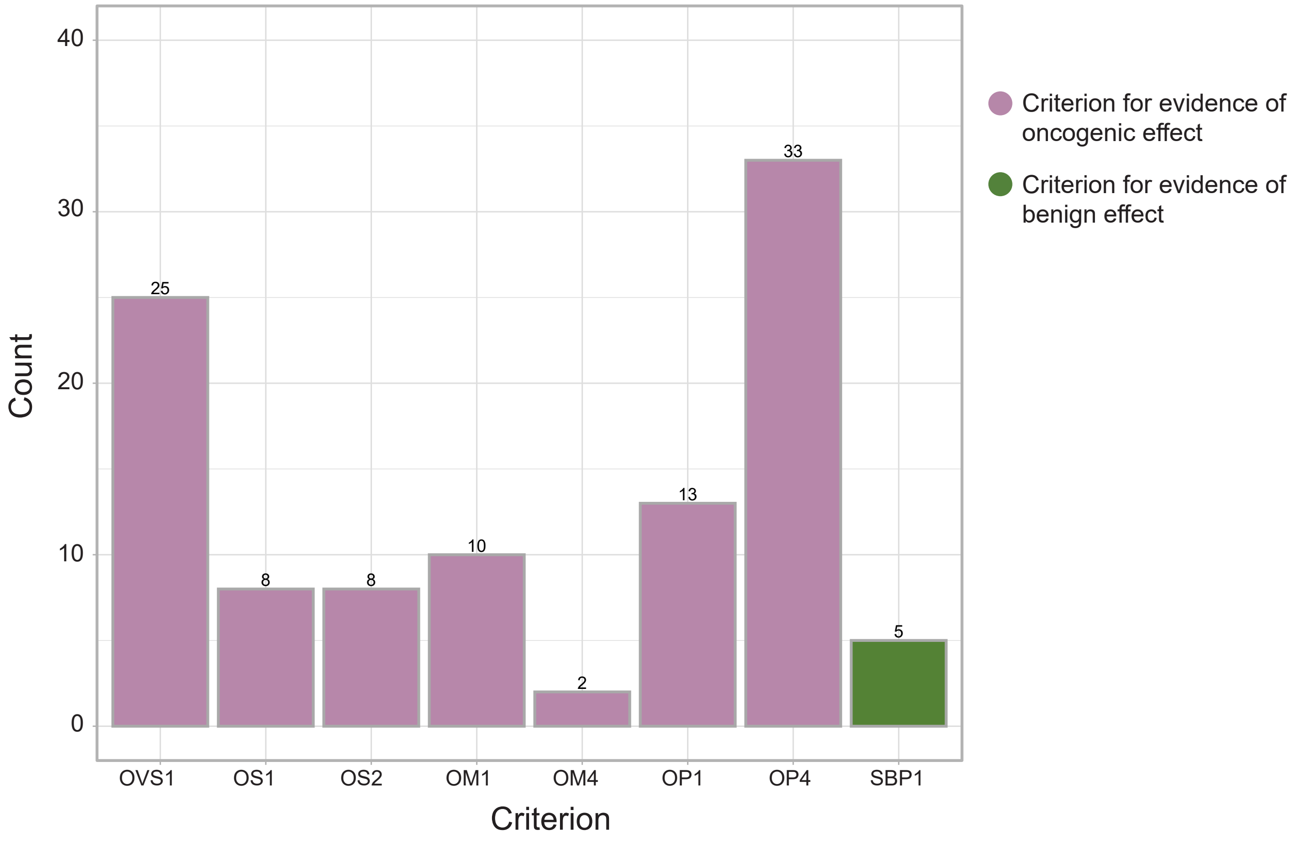

Supplement: Supplemental Figure S6 — Results on the variants re-assessed as oncogenic/likely oncogenic (O/LO) with agreement between expert and OncoVI classification. A: Dumbbell plot of the 33 variants classified as O/LO by both experts and OncoVI. Horizontal bars indicate the classification of the variants according to the standard operating procedure point-based system (ie, score ≥ 10: oncogenic; 6 ≤ score ≤ 9: likely oncogenic). B: Bar plot of the criteria triggered by OncoVI in the 33 variants classified as O/LO by both experts and OncoVI. Criteria are sorted according to decreasing corresponding points: OVS1, oncogenic very strong-1 (8 points); OS1, oncogenic strong-1 (4 points); OS2, oncogenic strong-2 (4 points); OM1, oncogenic moderate-1 (2 points); OM4, oncogenic moderate-4 (2 points); OP1, oncogenic supporting-1 (1 point); OP4, oncogenic supporting-4 (1 point); SBP1, somatic benign supporting-1 (–1 point). [file mmc6.pdf]
