# Supplementary material for: Oncogenicity Variant Interpreter (OncoVI) Supports Harmonized Somatic Variant Interpretation in Precision Oncology
Source: J Mol Diagn. 2026 Apr 3;28(6):469–84. doi: 10.1016/j.jmoldx.2026.03.004 (PMC13269341; doi:10.1016/j.jmoldx.2026.03.004)

Supp.Figure 7

A

Scores of the VUS variants with agreement in OncoVI and expert classification (n=75)

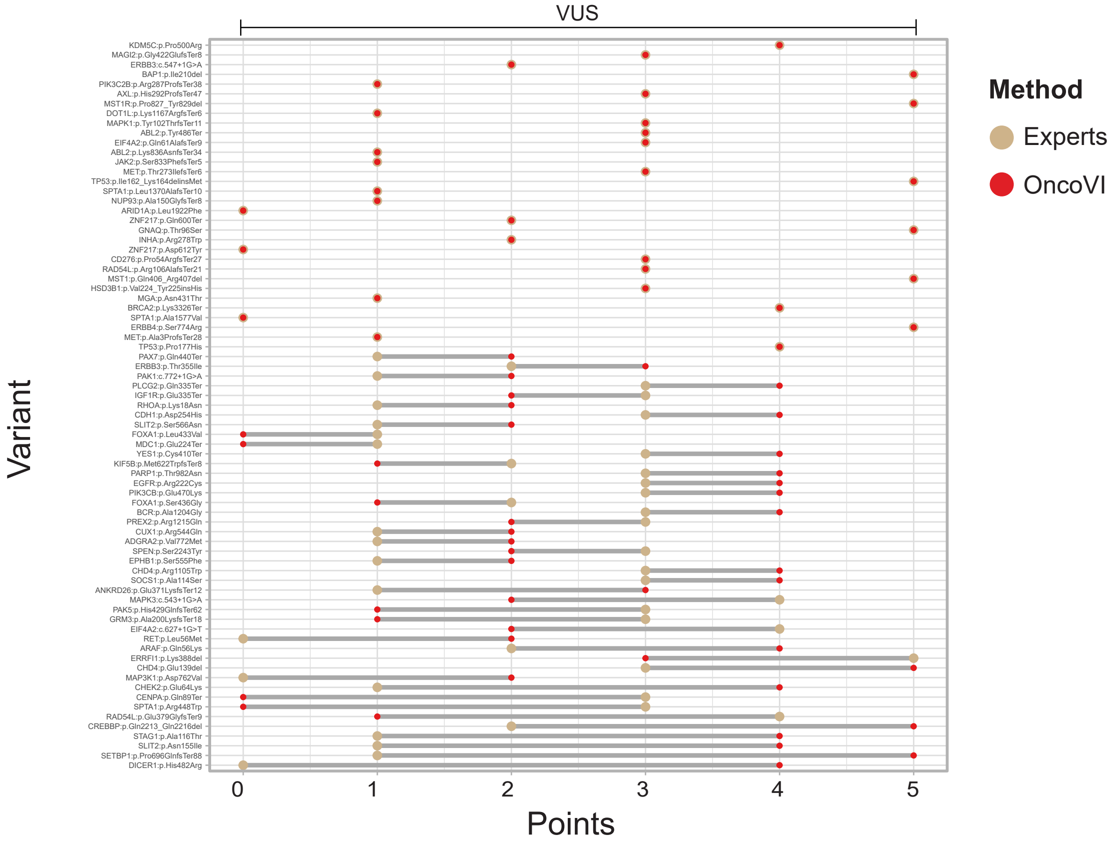

B

Criteria of the VUS variants with agreement in OncoVI and expert classification (n=75)

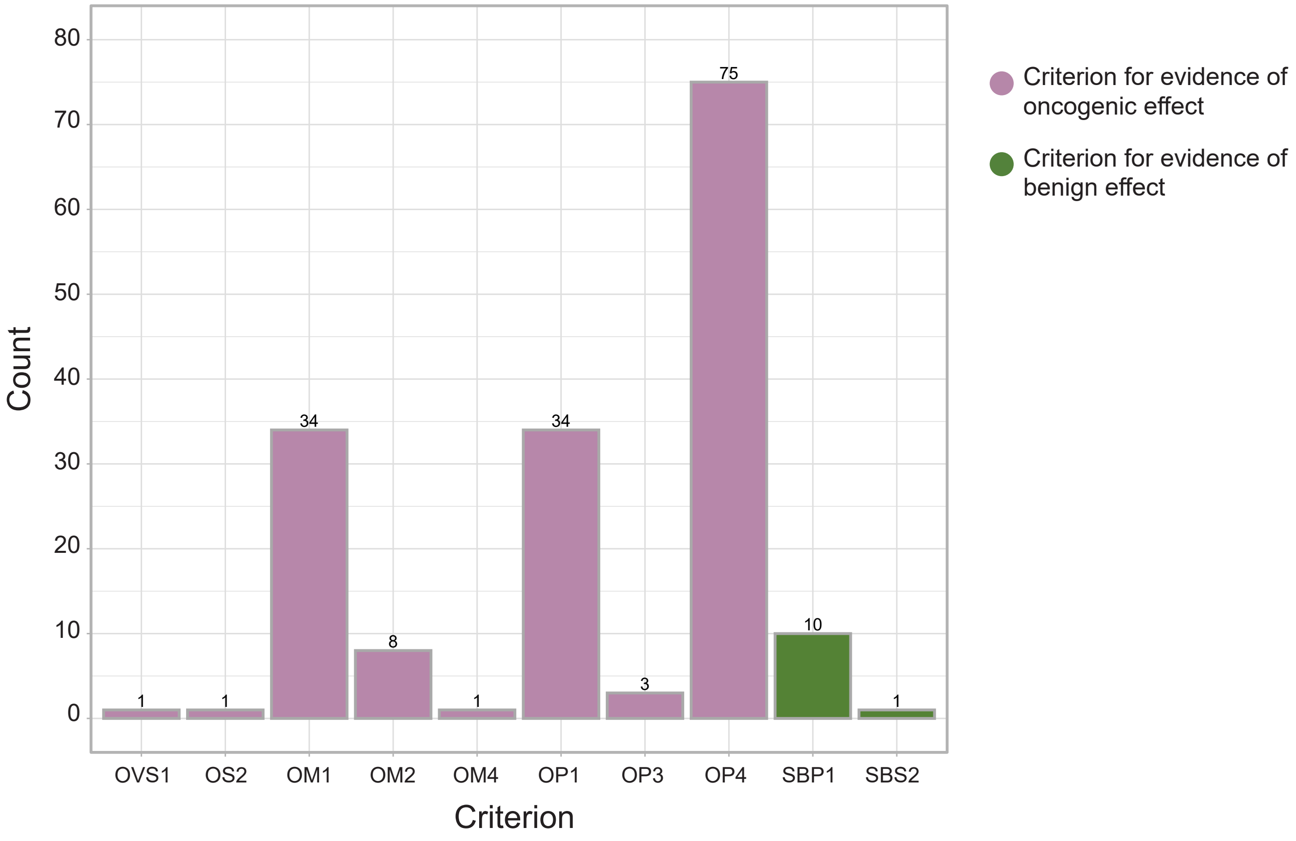

Supplement: Supplemental Figure S7 — Results on the variants re-assessed as variant of uncertain significance (VUS) with agreement between expert and OncoVI classification. A: Dumbbell plot of the 75 variants classified as VUS by both experts and OncoVI. Horizontal bars indicate the classification of the variants according to the standard operating procedure point-based system (ie, 0 ≤ score ≤ 5: VUS). B: Bar plot of the criteria triggered by OncoVI in the 75 variants classified as VUS by both experts and OncoVI. Criteria are sorted according to decreasing corresponding points: OVS1, oncogenic very strong-1 (8 points); OS2, oncogenic strong-2 (4 points); OM1, oncogenic moderate-1 (2 points); OM2, oncogenic moderate-2 (2 points); OM4, oncogenic moderate-4 (2 points); OP1, oncogenic supporting-1 (1 point); OP3, oncogenic supporting-3 (1 point); OP4, oncogenic supporting-4 (1 point); SBP1, somatic benign supporting-1 (–1 point); SBS2, somatic benign strong-2 (–4 points). [file mmc7.pdf]
