# Supplementary figures and images for: Oncogenicity Variant Interpreter (OncoVI) Supports Harmonized Somatic Variant Interpretation in Precision Oncology
Source: J Mol Diagn. 2026 Apr 3;28(6):469–84. doi: 10.1016/j.jmoldx.2026.03.004 (PMC13269341; doi:10.1016/j.jmoldx.2026.03.004)

# Supp.Figure 8

Re-assessed MTB variants with agreement between OncoVI and experts (n=108)

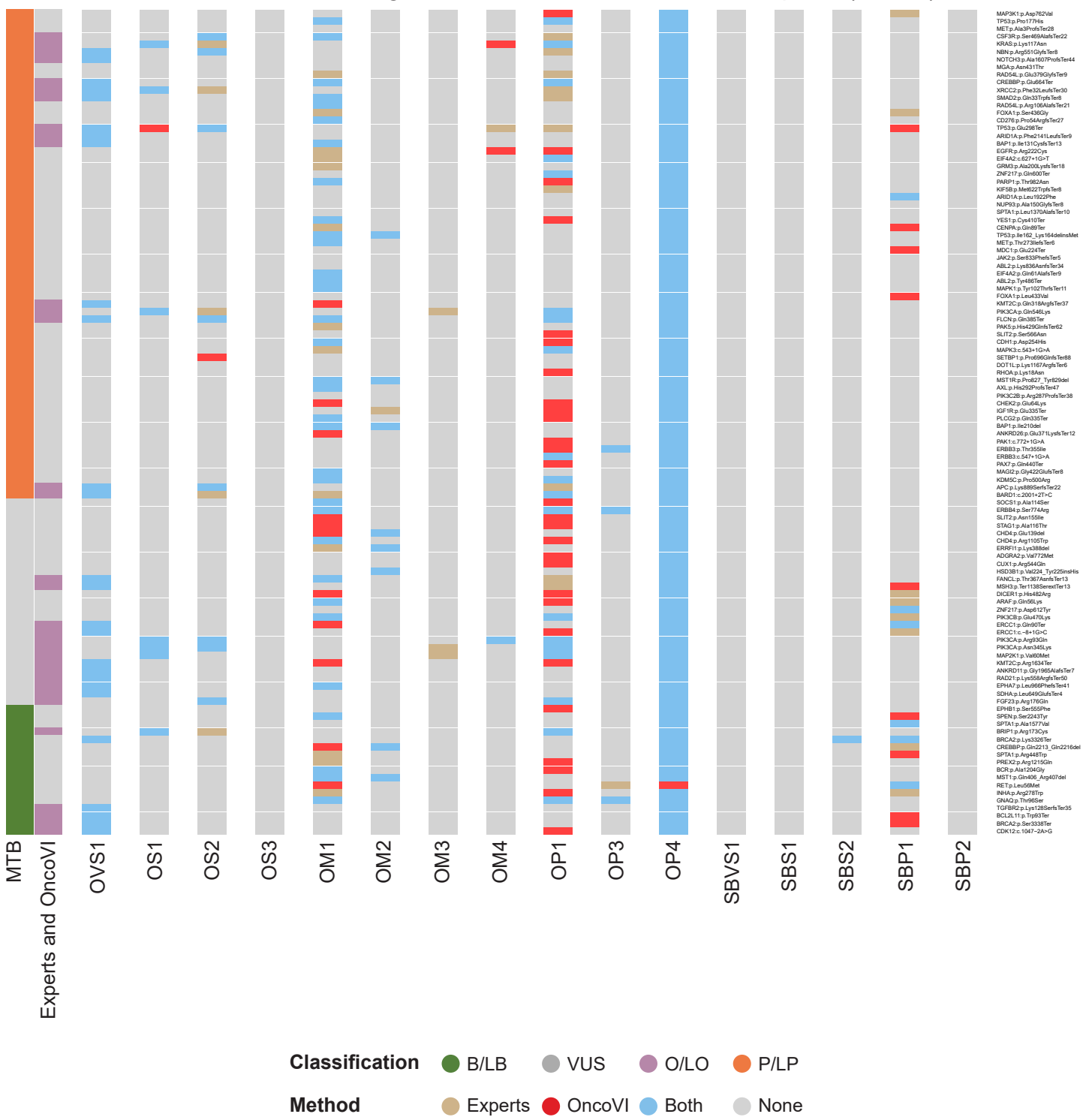

Supplement: Supplemental Figure S8 — Re-assessed Molecular Tumor Board (MTB) variants with agreement between expert and OncoVI classification. Each row corresponds to 1 of the 108 variants with agreement between expert and OncoVI classification, and each column corresponds to an assessed criterion. The color of the cell indicates whether a criterion was triggered by the experts only (dark gold), OncoVI only (red), both (light blue), or none of the two (gray). The bar plot on the left indicates the classification of each variant according to the MTB, OncoVI, and expert assessment. B/LB, benign/likely benign; O/LO, oncogenic/likely oncogenic; OM1, oncogenic moderate-1 (2 points); OM2, oncogenic moderate-2 (2 points); OM3, oncogenic moderate-3 (2 points); OM4, oncogenic moderate-4 (2 points); OP1, oncogenic supporting-1 (1 point); OP3, oncogenic supporting-3 (1 point); OP4, oncogenic supporting-4 (1 point); OS1, oncogenic strong-1 (4 points); OS2, oncogenic strong-2 (4 points); OS3, oncogenic strong-3 (4 points); OVS1, oncogenic very strong-1 (8 points); P/LP, pathogenic/likely pathogenic; SBS1, somatic benign strong-1 (–4 points); SBS2, somatic benign strong-2 (–4 points); SBP1, somatic benign supporting-1 (–1 point); SBP2, somatic benign supporting-2 (–1 point); SBVS1, somatic benign very strong-1 (–8 points); VUS, variant of uncertain significance. [file mmc8.pdf]

# Carta *et al.*, Supp.Figure 9

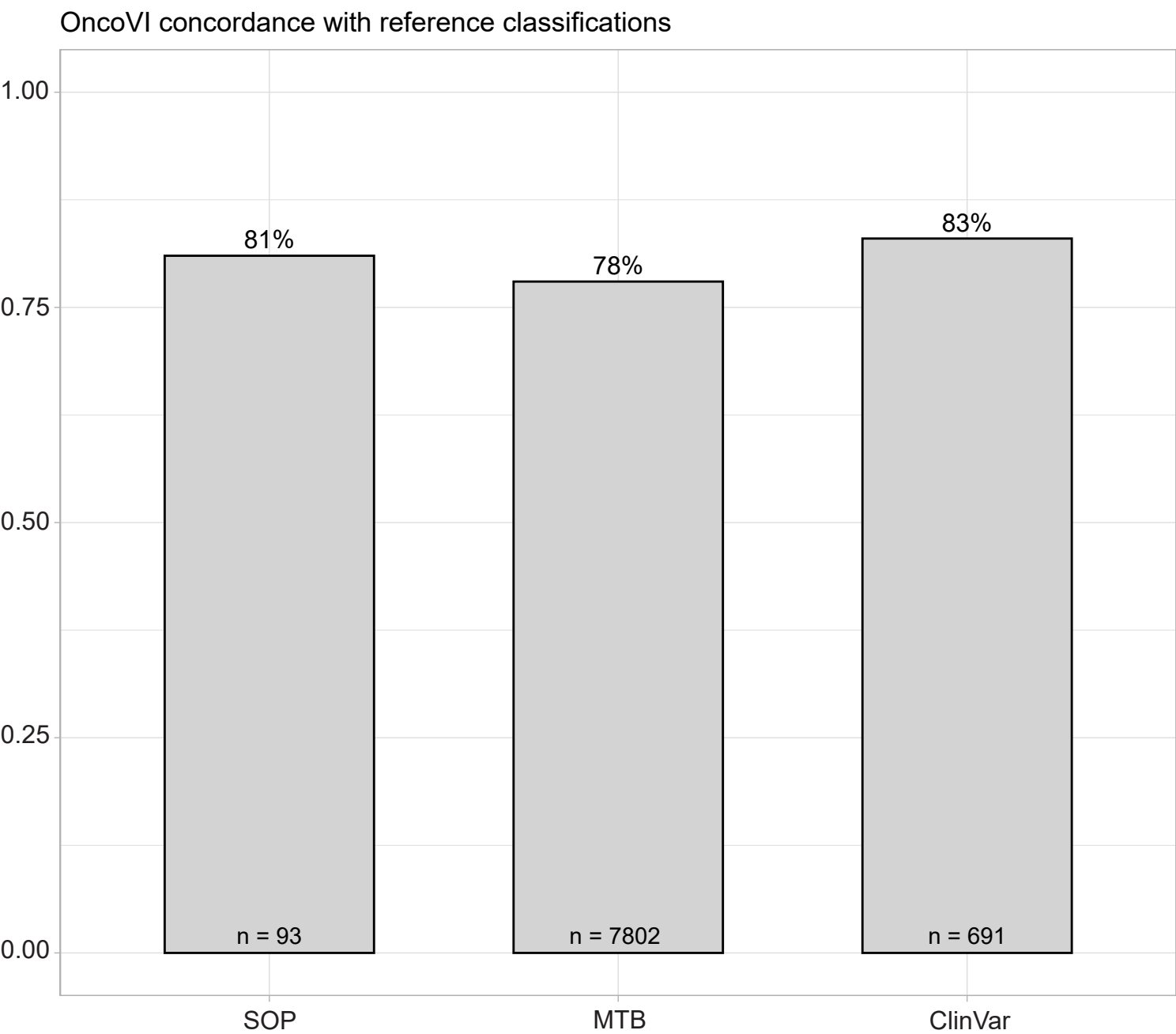

Supplement: Supplemental Figure S9 — OncoVI concordance with reference classification. Schematic summary of the three data sets used to evaluate OncoVI performance: the standard operating procedure (SOP) data set, the real-world Molecular Tumor Board (MTB) data set, and the ClinVar data set. For each data set, the number of analyzed variants and the concordance between OncoVI oncogenicity classification and the corresponding reference classification are shown. [file mmc9.pdf]
